# Genome-wide association study of facial morphology reveals novel associations with *FREM1* and *PARK2*

**DOI:** 10.1371/journal.pone.0176566

**Published:** 2017-04-25

**Authors:** Myoung Keun Lee, John R. Shaffer, Elizabeth J. Leslie, Ekaterina Orlova, Jenna C. Carlson, Eleanor Feingold, Mary L. Marazita, Seth M. Weinberg

**Affiliations:** 1 Center for Craniofacial and Dental Genetics, Department of Oral Biology, University of Pittsburgh, Pittsburgh, Pennsylvania, United States of America; 2 Department of Human Genetics, Graduate School of Public Health, University of Pittsburgh, Pittsburgh, Pennsylvania, United States of America; 3 Department of Biostatistics, Graduate School of Public Health, University of Pittsburgh, Pittsburgh, Pennsylvania, United States of America; 4 Clinical and Translational Science Institute, School of Medicine, University of Pittsburgh, Pittsburgh, Pennsylvania, United States of America; 5 Department of Psychiatry, School of Medicine, University of Pittsburgh, Pittsburgh, Pennsylvania, United States of America; 6 Department of Anthropology, University of Pittsburgh, Pittsburgh, Pennsylvania, United States of America; University of North Carolina at Chapel Hill, UNITED STATES

## Abstract

Several studies have now shown evidence of association between common genetic variants and quantitative facial traits in humans. The reported associations generally involve simple univariate measures and likely represent only a small fraction of the genetic loci influencing facial morphology. In this study, we applied factor analysis to a set of 276 facial linear distances derived from 3D facial surface images of 2187 unrelated individuals of European ancestry. We retained 23 facial factors, which we then tested for genetic associations using a genome-wide panel of 10,677,593 single nucleotide polymorphisms (SNPs). In total, we identified genome-wide significant (p < 5 × 10^−8^) associations in three regions, including two that are novel: one involving measures of midface height at 6q26 within an intron of *PARK2* (lead SNP rs9456748; p = 4.99 × 10^−8^) and another involving measures of central upper lip height at 9p22 within *FREM1* (lead SNP rs72713618; p = 2.02 × 10^−8^). In both cases, the genetic association was stronger with the composite facial factor phenotype than with any of the individual linear distances that comprise those factors. While the biological role of *PARK2* in the craniofacial complex is currently unclear, there is evidence from both mouse models and Mendelian syndromes that *FREM1* may influence facial variation. These results highlight the potential value of data-driven multivariate phenotyping for genetic studies of human facial morphology.

## Introduction

A number of studies have reported associations between genetic variants and normal-range variation in facial morphology. These include candidate gene studies focusing on a small number of genetic loci chosen based on their known roles in craniofacial development or in genetic syndromes [1–3] and genome-wide association studies (GWASs) that examine millions of genetic polymorphisms [4–8]. Such findings are anticipated by twin and family studies demonstrating the heritability of facial features. Notable findings include associations with *PAX3* and nasal root morphology in two independent studies [4,5]. More recently, a GWAS of nearly 6000 admixed South Americans revealed associations with nasal shape, implicating *DCHS2*, *RUNX2*, *GLI3*, *PAX1* and *EDAR* [6]. Another recent GWAS by our group [7] identified seven genetic associations in a European-derived cohort from the US involving 3D linear distance measures of orbital, nasal, and cranial base breadth and nasal projection, with associated loci harboring numerous genes involved in craniofacial syndromes (e.g., *ALX3*). Of interest, this study observed the same association between soft-tissue nasal width and *PAX1* reported by Adhikari et al. [6].

Prior association studies have used several diverse approaches to generate and test facial shape phenotypes. There is currently no agreement on the optimal phenotyping strategy. The variety of different measures and approaches used in prior studies makes it difficult to compare results and may partly explain the lack of replication across studies. To date, univariate tests involving simple linear distances or qualitatively graded facial features have generally shown the greatest success in GWAS designs. Such measures are often correlated, however, as the human craniofacial complex shows strong evidence of morphological integration [9]. The pattern of covariation observed among facial measures is thought to arise out of common developmental processes that drive morphogenesis and growth [10,11]. Approaches to phenotyping designed to capture this covariance structure offer an alternative and promising strategy to investigate the genetic basis of human facial variation. Unfortunately, the use of such methods in GWAS has had limited success to date. Paternoster et al. [4] applied factor analysis to a set of linear distances and landmark coordinate vectors, while Liu et al. [5] based their GWAS on principal components of shape derived from facial landmark coordinate data. Neither of these studies detected genome-wide significant associations based on the phenotypes derived. In both cases, however, only a small number of facial variables were included in the analyses, potentially rendering the extracted factors/components insufficient to capture key aspects of facial morphology.

To overcome some of these limitations, we used factor analysis (e.g., a method of pattern exaction that models correlated observed variables as linear combinations of unobserved latent variables) to derive composite measures of facial morphology based on a large number of traits in a well-characterized cohort of US individuals of European ancestry. Specifically, we applied factor analysis to a set of 276 facial linear distances derived from 3D facial surface images and then tested the resulting composite phenotypes for genetic associations using a genome-wide panel of single nucleotide polymorphisms (SNPs).

## Materials and methods

### Study sample

Our study sample was comprised of 2187 unrelated self-described White individuals of European ancestry from the United States (833 males and 1354 females). Participants were recruited at research centers in Pittsburgh, Seattle, Houston and Iowa City as part of the FaceBase Consortium’s 3D Facial Norms dataset [12]. Participants ranged from three to 40 years of age (mean age was 22.5 years). Exclusion criteria included a personal history of facial trauma, facial reconstructive or plastic surgery, orthognathic/jaw surgery or jaw advancement, facial prosthetics or implants, and any palsy, stroke or neurologic condition affecting the face. In addition, participants were excluded if they had a personal or family history of any facial anomaly or birth defect, or a personal or family history of any syndrome or congenital condition known to affect the head or face. Institutional review board (IRB) approval was obtained at each recruitment site and all participants gave their written informed consent prior to participation; for children, written consent was obtained from a parent or legal guardian (University of Pittsburgh IRB #PRO09060553 and #RB0405013; UT Health Committee for the Protection of Human Subjects #HSC-DB-09-0508; Seattle Children’s IRB #12107; University of Iowa Human Subjects Office/IRB #200912764 and #200710721).

### Facial imaging and landmarking

3D facial surfaces were captured via digital stereophotogrammetry (3dMD imaging systems, Atlanta, GA) using published protocols [12,13]. Twenty-four standard facial soft-tissue landmarks (S1 Fig) were collected on each 3D facial surface and the xyz coordinate locations saved. These landmarks were chosen because they exhibit high levels of precision when identified on 3D facial surface scans, while simultaneously providing adequate facial coverage. Weinberg et al. [12] have provided detailed descriptions on the data quality checking and landmarking error analysis for this dataset.

### Phenotyping approach

A set of 276 facial measurements was generated for each individual in the dataset. These 276 measurements represent every possible unique Euclidean distance calculated between the 3D coordinates of our set of 24 facial landmarks. These distances were calculated using the program WinEDMA v1.0.1 [14]. Factor analysis, a method of constructing unobserved latent variables from correlated observed variables, was applied to the set of linear distances. In brief, factor analysis works by modeling observed variables as linear combinations of hypothesized and unobserved latent variables plus error terms, and is intended for datasets where a large number of observed variables are thought to be governed by a smaller number of underlying processes. In this regard, factor analysis may be appropriate to the investigation of facial shape. Given that many of the 276 distances capture similar, but not identical, aspects of facial morphology, an advantage of factor analysis is that it can identify sets of facial measures that exhibit strong patterns of covariance allowing these correlated traits to be analyzed collectively (i.e., as factors). This approach can be useful for gene mapping if the covariation reflected in these factors is due, at least in part, to the effect of genes. Because numerous extrinsic variables are known to affect facial morphology, prior to the factor analysis each of the 276 linear distances was adjusted for the effects of sex, age, age^2^, height, and weight using linear regression. This resulted in 276 adjusted phenotypes (i.e., residuals), which were entered into the factor analysis. Visual inspection of the scree plot and parallel analysis were used to determine the number of factors to retain. Specifically, factors were retained if the observed eigenvalues from the correlation matrix were greater than the mean obtained from random uncorrelated data [15]. To aid with interpretation of the factors, varimax rotation was applied. The factor analysis was performed in SAS 9.4 (SAS, Cary, NC, USA).

### Genotyping, imputation, and population structure

Genotyping and data cleaning was performed as previously described [7]. In brief, DNA extracted from saliva samples was genotyped for 964,193 SNPs on the Illumina (San Diego, CA) OmniExpress+Exome v1.2 array plus 4,322 custom SNPs. HapMap control samples (N = 72) were genotyped alongside study participants for quality assurance. Standard data cleaning procedures and quality assurance analyses were performed as describe previously [16]. These included interrogating samples for genetic sex, chromosomal anomalies, relatedness among participants, missing call rate, and batch effects, and interrogating SNPs for missing call rate, discordance between duplicate samples, Mendelian errors (as measured in HapMap control parent-offspring trios), Hardy-Weinberg equilibrium, and differences in allele frequency and heterozygosity between sexes. Genotyping was performed by the Center for Inherited Disease Research (CIDR). Data cleaning was performed in collaboration with the University of Washington Genetics Coordinating Center (UWGCC).

Imputation was performed to capture information on unobserved SNPs as well as sporadically missing genotypes among genotyped SNPs, using all haplotypes from the 1000 Genomes Project [17] Phase 3 reference panel (Phase 1 for X chromosome because Phase 3 was not released for the X chromosome at the time of analysis). First, pre-phasing was performed in SHAPEIT2 [18], and then imputation of 34,985,077 variants was performed in IMPUTE2 [19,20]. Imputed SNPs with INFO scores less than 0.5 were filtered out of the analysis. For imputed SNPs retained in the study, imputed genotypes were included in analyses only if the genotype probability for a given variant in a given participant was greater than 50%. Average INFO scores were 0.97, 0.93, and 0.87 for SNPs with minor allele frequencies (MAF) greater than 5%, 2.5% to 5%, and less 2.5%, respectively.

Population structure was assessed with principal component analysis using 96,700 autosomal SNPs pruned from the total panel based on call rate (> 95%), MAF (> 0.05), and LD (pairwise r^2^ < 0.1 in a sliding window of 10 Mb). Linear regression, testing the association between each principal component (PC) and each SNP in the genome, confirmed that none of the first 20 PCs of ancestry were due to local variation in specific genomic regions. Based on the scree plot and joint distributions, we determined that four PCs were sufficient for capturing the population structure. Joint distributions of the four PCs, and their joint distributions with the 23 factors, are provided in the S2 Fig.

### Genetic association analyses

The genetic association analyses were performed using PLINK [21]. Linear models were used to test for genetic association between each of the extracted factors and each SNP, under an additive genetic model, while simultaneously adjusting for the first four principal components of ancestry. On the X chromosome, genotypes in hemizygous males were coded 0/2 so they are on the same scale as 0/1/2 females. To appropriately model SNP effects, the minor allele was required to be present in at least 30 participants, corresponding to MAF threshold of 0.6%. The final number of genotyped SNPs after minor allele filtering was 659,955. The final number of imputed and genotyped SNPs available for analysis was 10,677,593.

As customary in the field, we accounted for the issue of multiple testing by considering p < 5 × 10^−8^ (i.e., Bonferroni correction for 1 million tests) the threshold for genome-wide statistical significance. Because this threshold is conservative and the overall approach here can be considered hypothesis-generating, we also reported “suggestive” evidence of association at p < 5 × 10^−6^ in the Supplemental Material. In order to account for multiple GWAS scans corresponding to the 23 factors, we consider p < 2.17 × 10^−9^ (i.e., Bonferroni correction for 23 million tests) the strict threshold for study-wide significance.

## Results

### Factor analysis of facial morphology

A total of 276 factors were extracted, 23 of which were retained. These 23 factors captured approximately 94% of the variation. The first four factors alone captured over 54% of the variation. As expected, moving from the first to the last factor revealed a general shift away from more global aspects of facial variation to more localized regional effects. Factor 1, for example, described 36% of the variation and involved multiple measures capturing the overall horizontal breadth of the face. In contrast, factor 21explained 0.6% of the variation and involved measurements comprised of only two landmarks on the nasal alae. Table 1 provides an interpretation of each factor based on the specific measurements and landmarks involved. Many of the factors captured aspects of facial variation commonly measured with clinical facial anthropometry. For example, factors 3, 4, 6, 9 and 17 all described different aspects of vertical facial height, whereas factors 1, 5, 7, 10, 14 and 15 captured commonly measured aspects of facial breadth. In contrast, other factors (e.g., 2, 8, 13, and 21) captured complex aspects of facial variation. Factor 8, for example, described the horizontal and vertical position of the exocanthion landmarks relative to more centrally located structures, which may relate, in part, to the inclination of the palpebral fissures. Six of the 23 factors could not be easily interpreted due to weak loadings of many linear distances; these six factors each explained 1% or less of the variation. The loading of each distance on each factor is provided in S1 Table.

**Table 1:** Description of the 23 extracted factors describing facial morphology.

Factor	Variation explained (%)	Cumulative variation (%)	Description	Key landmarks^1^
1	36.2	36.2	Breadth of the lateral portion of the upper face	Tragion
2	10.2	46.4	Vertical position of the orbits relative to the midface	Endocanthion & exocanthion
3	8.4	54.8	Length of the philtrum	Crista philtri & labiale superius
4	6.2	60.9	Facial height related to the vertical position of gnathion	Gnathion
5	5.3	66.2	Width of the mouth relative to the central midface	Chelion
6	3.8	70.1	Height of the vermilion lower lip	Labiale inferius
7	3.6	73.6	Width of the cartilaginous portion of the nose	Alare, alar curvature point, subalare, & subnasale
8	2.6	76.2	Orbital inclination due to the vertical and horizontal position of exocanthion	Exocanthion
9	2.5	78.7	Facial height related to the vertical position of nasion	Nasion
10	2.1	80.9	Width of the nasal floor	Subalare
11	1.7	82.5	Projection of the nose	Pronasale
12	1.5	84.0	Vertical position of the sublabial sulcus relative to the central midface	Sublabiale
13	1.4	85.5	Vertical position of the alar curvature point relative to the upper lip	Alar curvature point
14	1.1	86.6	Intercanthal width	Endocanthion
15	1.1	87.6	Philtrum width	Crista philtri
16	1.0	88.7	Not defined *	
17	.09	89.6	Height of the vermilion upper lip	Stomion
18	.08	90.3	Not defined *	
19	.07	91.1	Not defined *	
20	.07	91.7	Not defined *	
21	.06	92.4	Depth of the nasal alae	Alare & Alar curvature point
22	.06	93.0	Not defined *	
23	.06	93.6	Not defined *	

^1^ see S1 Fig for landmark locations

*No measures showed meaningful loading on these factors

### Genetic association analysis

**Fig 1:**
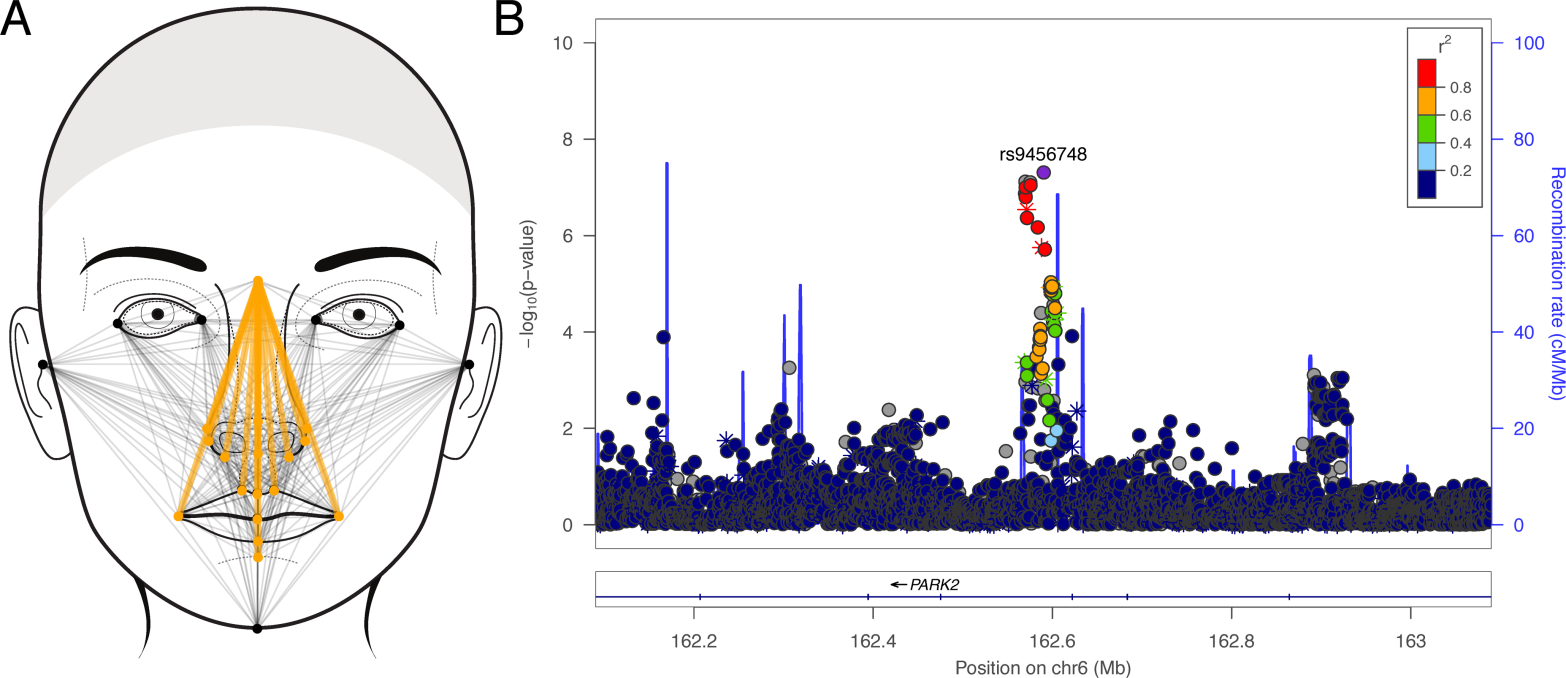
Phenotype and association results for facial factor 9. (A) Face showing the linear distances (in dark yellow) associated with factor 9; (B) LocusZoom plot showing the association (left y-axis; log10-transformed p-values) with factor 9. Genotyped SNPs are depicted by stars and imputed SNPs are depicted by circles. Shading of the points represents the linkage disequilibrium (r^2^, based on the 1000 Genomes Project Europeans; gray indicates unknown LD) between each SNP and the top SNP, indicated by purple shading. The blue overlay shows the recombination rate (right y-axis). Positions of genes are shown below the plot. Note, gray points near the lead SNP are insertion-deletion variants in high LD (r2 = 0.91 and 0.77) with the lead SNP in our cohort.

**Fig 2:**
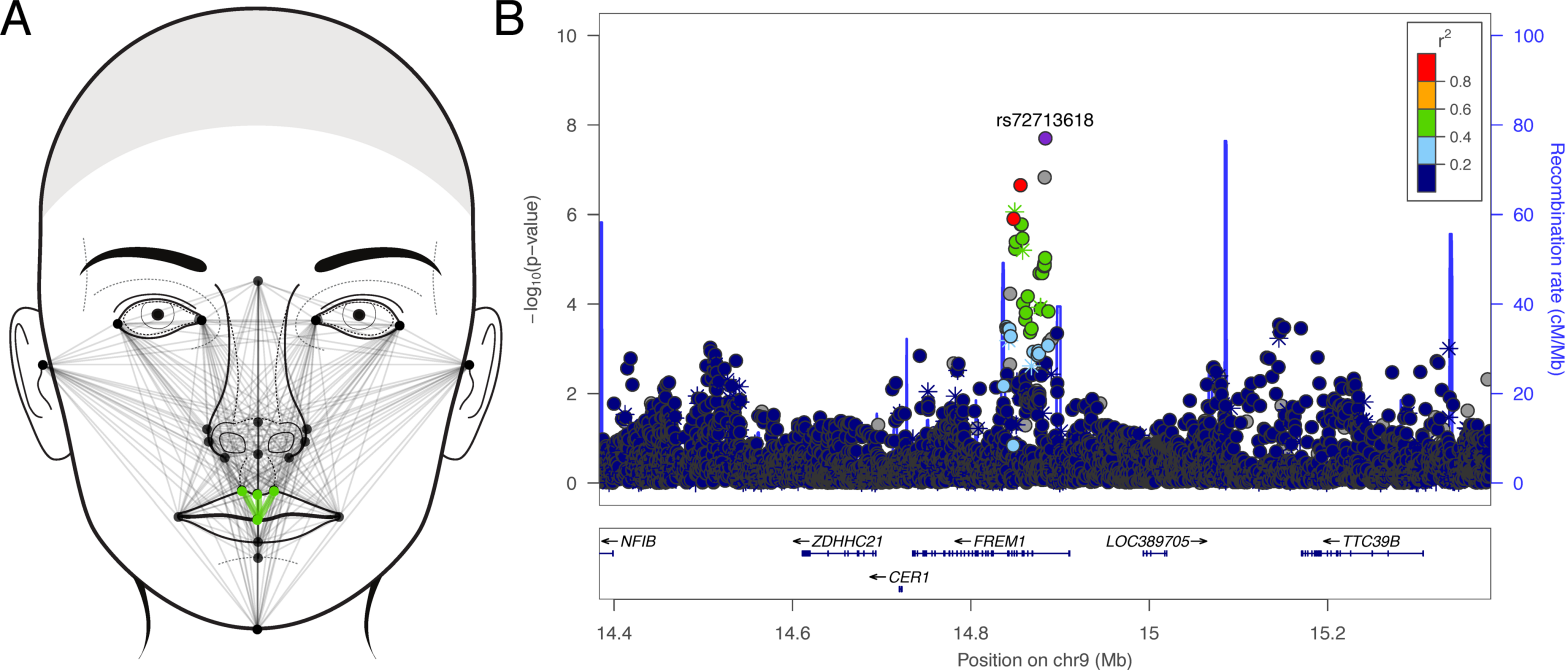
Phenotype and association results for facial factor 17. (A) Face showing the linear distances (in light green) associated with factor 17; (B) LocusZoom plot showing the association (left y-axis; log10-transformed p-values) with factor 17. Genotyped SNPs are depicted by stars and imputed SNPs are depicted by circles. Shading of the points represents the linkage disequilibrium (r^2^, based on the 1000 Genomes Project Europeans; gray indicates unknown LD) between each SNP and the top SNP, indicated by purple shading. The blue overlay shows the recombination rate (right y-axis). Positions of genes are shown below the plot. Note, the gray point near the lead SNP is an insertion-deletion variant in high LD (r2 = 0.82) with the lead SNP in our cohort.

**Fig 3:**
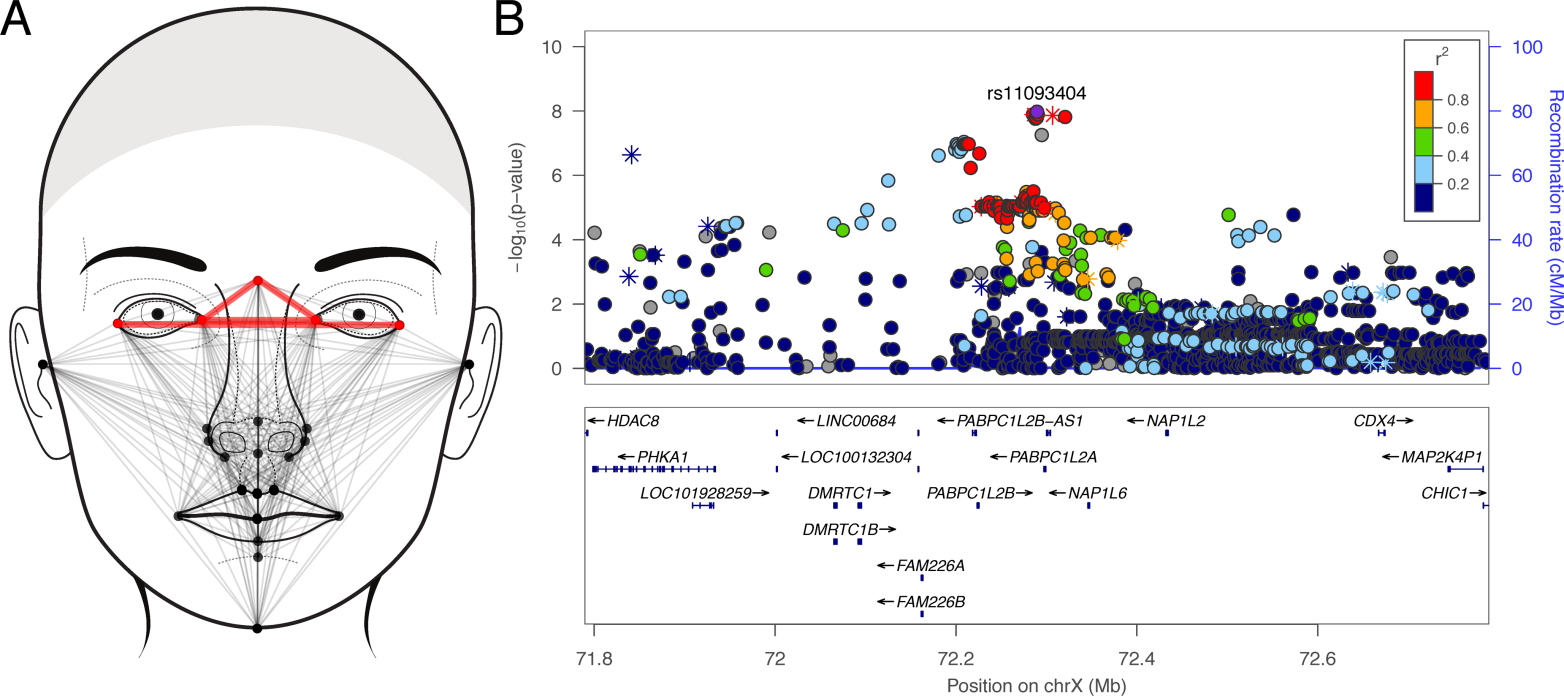
Phenotype and association results for facial factor 14. (A) Face showing the linear distances (in red) associated with factor 14; (B) LocusZoom plot showing the association (left y-axis; log10-transformed p-values) with factor 14. Genotyped SNPs are depicted by stars and imputed SNPs are depicted by circles. Shading of the points represents the linkage disequilibrium (r^2^, based on the 1000 Genomes Project Europeans; gray indicates unknown LD) between each SNP and the top SNP, indicated by purple shading. The blue overlay shows the recombination rate (right y-axis). Positions of genes are shown below the plot. Note, the gray point near the lead SNP is an insertion-deletion variant in high LD (r2 = 0.97) with the lead SNP in our cohort.

Among the 23 facial morphology factors, we identified seven genome-wide significant associations (Table 2). At three loci (6q26, 9p22, and Xq13), multiple SNPs reached or approached genome-wide significance. Factor 9, which involved measures of facial height containing the landmark nasion, was associated with a locus on 6q26 (Fig 1A). The lead SNP (rs9456748; p = 4.99 × 10^−8^) was located within a narrow LD block within an intron of *PARK2*, a gene that spans 1.3Mb (Fig 1B). Factor 17, which involved measures of upper lip vermillion height (Fig 2A), was associated with a 100Kb region of 9p22 within *FREM1* (Fig 2B; lead SNP rs72713618; p = 2.02 × 10^−8^). Factor 14, which captured the horizontal spacing of the inner canthi of the orbits (Fig 3A), was associated with a region spanning 1Mb on Xq13 (Fig 3B; lead SNP rs11093404; p = 1.07 × 10^−8^). The four remaining association signals at 8q12 (factor 21; top SNP: rs113036800; p = 1.20 × 10^−8^), 12q24.2 (factor 3; top SNP: rs117438382; p = 3.68 × 10^−8^), 16p12.1 (factor 7; top SNP: rs62031988; p = 2.01 × 10^−8^), and Xp11.3 (factor 22; top SNP: rs138440928; p = 1.85 × 10^−8^) involved isolated imputed SNPs (S3 Fig) with imputation INFO score less than 0.9, and therefore we advocate caution in interpreting these. Manhattan plots for all the 23 factors are included in S4 Fig. Due to the fact that the cohort spans broad range of ages (3–40 years), as a sensitivity analysis, we reran association for our top hits in the subset of participants 16–40 years. Results (beta-coefficient and p-values) were not meaningfully different (see S2 Table).

**Table 2:** Genome-wide significant results for seven facial factors.

Trait	Lead SNP	Locus	BP	Minor allele ^a^	Major allele	MAF	n	Beta (SE)	p	INFO
Factor 3	rs117438382 ^b^	12q24.2	119414564	A	C	0.009	2184	0.887 (0.161)	3.76 × 10^−8^	0.659
Factor 7	rs62031988 ^b^	16p12.1	27017495	C	T	0.015	2185	0.700(0.124)	2.01 × 10^−8^	0.713
Factor 9	rs9456748	6q26	162590018	G	A	0.441	2187	0.165 (0.030)	4.99 × 10^−8^	0.99
Factor 14	rs11093404	Xq13	72289467	A	G	0.248	2187	0.175 (0.030)	1.07 × 10^−8^	0.997
Factor 17	rs72713618	9p22	14883254	A	G	0.021	2187	-0.592 (0.105)	2.02 × 10^−8^	0.98
Factor 21	rs113036800 ^b^	8q12	58449027	T	C	0.015	2187	0.691 (0.121)	1.20 × 10^−8^	0.839
Factor 22	rs138440928 ^b^	Xp11.3	44350343	A	C	0.011	2187	0.650 (0.115)	1.85 × 10^−8^	0.522

BP = Base position; MAF = Minor allele frequency; INFO = Info score

^a^ The minor allele is the risk allele

^b^ the lead SNP is the only variant showing evidence of association at this locus

For three of the seven genome-wide significant signals, we observed that genetic associations with the factors were stronger than with any of the constituent linear distances that comprised the factors. For example, the association between rs9456748 and factor 9 was 4.99 × 10^−8^, whereas the p-values for association tests with the 16 constituent linear distances ranged from 2.27 × 10^−4^ to 1.75 × 10^−6^. These results are shown in S3 Table. We also observed a large number of “suggestive” signals (p < 5 × 10^−6^), which are detailed in S4 Table.

## Discussion

In this study, we performed a GWAS of composite facial traits in a sample of 2187 unrelated healthy individuals. To derive these traits, we applied factor analysis to 276 facial linear distances calculated between the 3D coordinates of 24 facial surface landmarks. Analysis of 23 distinct factors, accounting for 94% of the variation, revealed seven genetic associations exceeding the strict threshold for genome-wide statistical significance (p < 5 × 10^−8^).

We observed a novel association between SNPs in *FREM1* and a factor capturing the height of the central portion of the upper lip. *FREM1* encodes a basement membrane protein involved in epithelial-mesenchymal transformations and maintenance of epidermal adhesion [22]. *Frem1* is expressed in several murine craniofacial structures including the eyelids, ears, forehead, and midface [22–24]. Of particular relevance for our reported phenotypic association, Alazami et al. [23] reported strong *Frem1* expression in the midline where the left and right medial nasal processes fuse. In humans, the medial nasal processes contribute to both the central portion of the nose but also the philtrum and central portion of the vermilion lip [25]. These are the same anatomical regions captured by factor 17 in our analysis (Fig 2A). In humans, mutations in *FREM1* result in several Mendelian conditions with affected midline or para-midline craniofacial features, including BNAR (bifid nose with or without anorectal and renal anomalies) syndrome [23], Manitoba oculotrichoanal syndrome [26], and trigonocephaly [24]. *Frem1* mutant mice have similar phenotypic features including reduced snout projection and a shorter philtrum [26]. These findings provide biological support for common variants in *FREM1* influencing normal variation in philtrum and central upper lip morphology in humans.

We observed a novel association between SNPs in the *PARK2* gene and a factor capturing aspects of midfacial height (Fig 1A). *PARK2* encodes a protein involved in proteasomal degradation and is primarily known for its role in juvenile-onset Parkinson disease, which is caused by homozygous point mutations or deletions in this gene [27]. However, *PARK2* also spans 1.3Mb on 6q26 resulting in association signals with multiple ostensibly unrelated phenotypes including disc degeneration [28], cholesterol levels [29], leprosy [30], and, from the present study, midfacial height. It is difficult to speculate how these associated SNPs contribute to facial morphology. The only evidence that *PARK2* is expressed in the face is a weak signal in the olfactory epithelium in TS22 mouse embryos [31]. Similarly, other genes within the same topological domain (e.g. *QKI* and *PDE10A*) do not exhibit strong craniofacial expression. Finally, this interval is largely devoid of chromatin signatures for regulatory elements and bioinformatic analysis of the top SNPs did not reveal compelling annotations. Despite a strong statistical signal at this locus, there is currently little biological evidence pointing toward a possible mechanism by which these SNPs influence midfacial height.

The other major association involved the Xq13 locus and factor 14, which involved the horizontal spacing of the inner canthi of the eyes (Fig 3A). Associated SNPs span a 1Mb interval that includes several genes, many of which have not been studied in detail. We recently identified an association between this locus and intercanthal distance using the same study sample [7]. In that study we suggested that the relevant gene might be *HDAC8*, which is associated with Cornelia de Lange syndrome–a condition characterized by hypertelorism. As is evident on the LocusZoom plot (Fig 3B), the *HDAC8* gene is located almost 500kb centromeric to the top SNP in the current analysis. Another interesting gene is *NAP1L2*, a nucleosome assembly protein required for neurulation, a developmental process that includes formation of neural crest cells [32]. Abnormalities in neural crest development cause several disorders with midline defects including frontonasal dysplasia and Waardenburg syndrome.

The other four associations on 12q24, 16p12.1, 8q12, and Xp11.3 were more difficult to interpret as they involved a limited number of imputed SNPs (S3 Fig). The association at 12q24.2 with factor 3 involved a single isolated and imputed SNP and no genes in the region were known to have a craniofacial function. The association at 16p12.1with factor 7 again involved only a handful of isolated imputed SNPs, although nearby gene *KDM8* may play a role in midface development [33]. The associations at 8q12 and Xp11.3 were with factors 21 and 22, respectively. These factors each explained very small proportions of the variation and showed little or no evidence of meaningful factor loadings (S1 Table). The associated SNP at Xp11.3 was upstream from *KDM6A*, mutations in which are known to cause Kabuki syndrome [34]. Thus, while these loci could contain relevant genes, deciphering their role in facial morphology will depend on independent confirmation of these results

For three of the results described above, including *FREM1* and *PARK2*, genetic associations with the factors were stronger than with any of the individual distance measures that comprised the factors. This suggests that factor analysis, at least in some instances, is better able to capture biologically relevant aspects of facial morphology compared with simple univariate distance measures. Because the factors are data-driven phenotypes reflecting the covariance structure of the human face, they are potentially less biased than conventional measures, which are selected *a priori* for reasons that may have little to do with biology. One explanation for the covariance within and among facial features is that the growth and development of the face is a highly coordinated process. This coordination may be driven in part by genes influencing, either directly or indirectly, one or more parts of the face. Factor analysis is one approach that allows us to leverage this genetically influenced covariance and examine its basis through genetic association analysis.

A major challenge in gene-mapping studies is making the jump from associated locus to causal variant. Though we describe biologically plausible candidates (e.g., *FREM1* and *PARK2*) based on physical proximity and known biology, the tasks of determining exactly which causal variant accounts for an association signal, which gene the variant impacts, and through what mechanism the variant acts, are difficult. Typically observational evidence, alone, is insufficient in identifying a putative causal allele, and additional experimental work is usually necessary to understand the mechanism. Therefore, more work is needed, beginning with replication of these genetic associations in independent cohorts, in order to truly understand the contributions of associated variants to human facial morphology.

A weakness of the current study was the lack of an appropriate cohort for independent replication. A persistent challenge in the area of human facial genetic studies is the lack of consistent phenotyping across existing cohorts, making replication difficult. The availability of automated facial landmarking methods may offer a potential solution to this problem in the future. Consistent phenotyping may also aid in exploring facial morphology across ancestry groups. Whereas the current study was limited to self-reported non-Hispanic whites, facial shape exhibits variation both within and between racial and ethnic population; therefore, future work may permit analyses that combine samples of diverse ancestry to investigate the commonalities and differences in the genetic architecture of facial morphology across populations.

Another limitation of this study was the sparse set of facial landmarks that can only capture limited information about complex facial features and cannot adequately capture the morphology of facial regions like the cheeks and forehead. Although 276 facial measures were included in our factor analysis, these linear distances were derived from just 24 facial landmarks, and represent only part of the dense information contained within 3D facial images. Moreover, factor analysis is only one possible method of analysis, and it ultimately seeks to reduce the dimensionality of the data, leading to loss of information. These limitations may be overcome through the development of phenotyping methods that better utilize the morphological richness contained within the full 3D facial surface [3].

In conclusion, we identified novel genetic associations with composite facial variation phenotypes, which were not observed in our previously GWAS of selected linear distances between facial landmarks. These results showcase the benefit of data-driven phenotyping for gene discovery of complex traits. Among the associated loci were genes, such as *FREM1* and *HDAC8*, with corroborating evidence for roles in facial variation based on human syndromes or model organisms. Other associations pointed to genes, such as *PARK2*, not previously implicated in facial variation. This study contributes to our understanding of the genetic basis of human facial variation and underscores the need for advances in phenotyping methods that capture the biologically relevant variation in human facial morphology.

## Supporting information

S1 Table The loading of all 276 linear distances on each of the 23 retained factors. (XLSX)

S2 Table Comparison of top associations in all participants and the subset of participants ages 16–40 years. (DOCX)

S3 Table P-values for the seven factors with significant associations compared to the individual linear distances that comprise those factors. (XLSX)

S4 Table All SNPs associated with the 23 factors at p < 5 × 10^−6^. (XLSX)

S1 Fig The location of the 24 standard facial landmarks used to generate the 276 linear distances. Landmarks are labeled as follows: n = nasion; prn = pronasale; sn = subnasale; ls = labiale superius; sto = stomion; li = labiale inferius; sl = sublabiale; gn = gnathion; en = endocanthion; ex = exocanthion; al = alare; ac = alar curvature point; sbal = subalare; cph = crista philtri; ch = chelion; and t = tragion. For bilateral landmarks, left and right indicated by _l and _r after the landmark abbreviation.
(PDF)

S2 Fig Joint distribution of the four PCs of ancestry and 23 factors. All pairwise combinations of Eigenvectors (EV; i.e., the values associated with each PC) and factors are depicted via scatterplots. Pearson correlation coefficient (r) and significance of the correlation (p) are indicated for each pair.
(PDF)

S3 Fig **LocusZoom plots showing genome-wide significant associations observed for Factor 3 (A), Factor 21 (B), and Factor 22 (C)**. LocusZoom plots show the association (left y-axis; log10-transformed p-values) with each factor. Genotyped SNPs are depicted by stars and imputed SNPs are depicted by circles. Shading of the points represents the linkage disequilibrium (r2, based on the 1000 Genomes Project Europeans) between each SNP and the top SNP, indicated by purple shading. The blue overlay shows the recombination rate (right y-axis). Positions of genes are shown below the plot.
(PDF)

S4 Fig Manhattan plots for the 23 factors showing all genotyped and imputed SNPs. Chromosomes are arranged in order along the x-axis. The y-axis shows the log base 10 p-value. Lines for p-value thresholds set at 5 x 10^−8^ for genome-wide significance and 5 x 10^−6^ for suggestive significance.
(PDF)

## References

[1] Coussens AK, van Daal A. Linkage disequilibrium analysis identifies an FGFR1 haplotype-tag SNP associated with normal variation in craniofacial shape. Genomics. 2005; 85:563–73. doi: 10.1016/j.ygeno.2005.02.002 15820308 10.1016/j.ygeno.2005.02.002

[2] Peng S, Tan J, Hu S, Zhou H, Guo J, Jin L, et al Detecting genetic association of common human facial morphological variation using high density 3D image registration. PLoS Comp Biol. 2013; 9:e1003375. 10.1371/journal.pcbi.1003375 PMC3854494 24339768

[3] Claes P, Liberton DK, Daniels K, Rosana KM, Quillen EE, Pearson LN, et al Modeling 3D facial shape from DNA. PLoS Genet. 2014; 10:e1004224 doi: 10.1371/journal.pgen.1004224 24651127 10.1371/journal.pgen.1004224 PMC3961191

[4] Paternoster L, Zhurov AI, Toma AM, Kemp JP, St Pourcain B, Timpson NJ, et al Genome-wide associ- ation study of three-dimensional facial morphology identifies a variant in PAX3 associated with nasion position. Am J Hum Genet. 2012; 90:478–485. doi: 10.1016/j.ajhg.2011.12.021 22341974 10.1016/j.ajhg.2011.12.021 PMC3309180

[5] Liu F, van der Lijn F, Schurmann C, Zhu G, Chakravarty MM, Hysi PG, et al A genome-wide associa- tion study identifies five loci influencing facial morphology in Europeans. PLoS Genet. 2012; 8: e1002932 doi: 10.1371/journal.pgen.1002932 23028347 10.1371/journal.pgen.1002932 PMC3441666

[6] Adhikari K, Fuentes-Guajardo M, Quinto-Sánchez M, Mendoza-Revilla J, Camilo Chacón-Duque J, Acuña-Alonzo V, et al A genome-wide association scan implicates DCHS2, RUNX2, GLI3, PAX1 and EDAR in human facial variation. Nat Commun. 2016; 7:11616 doi: 10.1038/ncomms11616 27193062 10.1038/ncomms11616 PMC4874031

[7] Shaffer JR, Orlova E, Lee MK, Leslie EJ, Raffensperger ZD, Heike CL, et al Genome-wide association study reveals multiple loci influencing normal human facial morphology. PLoS Genet. 2016; 12:e1006149 doi: 10.1371/journal.pgen.1006149 27560520 10.1371/journal.pgen.1006149 PMC4999139

[8] Cole JB, Manyama M, Kimwaga E, Mathayo J, Larson JR, Liberton DK, et al Genomewide association study of African children Identifies association of SCHIP1 and PDE8A with facial size and shape. PLoS Genet. 2016; 12:e1006174 doi: 10.1371/journal.pgen.1006174 27560698 10.1371/journal.pgen.1006174 PMC4999243

[9] Bastir M, Rosas A. Correlated variation between the lateral basicranium and the face: a geometric morphometric study in different human groups. Arch Oral Biol. 2006; 51:814–24. doi: 10.1016/j.archoralbio.2006.03.009 16681992 10.1016/j.archoralbio.2006.03.009

[10] Hallgrímsson B, Lieberman DE, Liu W, Ford-Hutchinson AF, Jirik FR. Epigenetic interactions and the structure of phenotypic variation in the cranium. Evol Dev. 2007; 9:76–91. doi: 10.1111/j.1525-142X.2006.00139.x 17227368 10.1111/j.1525-142X.2006.00139.x

[11] Parsons TE, Schmidt EJ, Boughner JC, Jamniczky HA, Marcucio RS, Hallgrímsson B. Epigenetic integration of the developing brain and face. Dev Dyn. 2011; 240:2233–44. doi: 10.1002/dvdy.22729 21901785 10.1002/dvdy.22729 PMC3246636

[12] Weinberg SM, Raffensperger ZD, Kesterke MJ, Heike CL, Cunningham ML, Hecht JT, et al The 3D Facial Norms Database: Part 1. A web-based craniofacial anthropometric and image repository for the clinical and research community. Cleft Palate Craniofac J. 2016;in press. 10.1597/15-199 PMC4841760 26492185

[13] Heike CL, Upson K, Stuhaug E, Weinberg SM. 3D digital stereophotogrammetry: A practical guide to facial image acquisition. Head Face Med. 2010; 6:18 doi: 10.1186/1746-160X-6-18 20667081 10.1186/1746-160X-6-18 PMC2920242

[14] Cole TM. WinEDMA: Software for Euclidean Distance Matrix Analysis. University of Missouri-Kansas City School of Medicine, Kansas City, MO; 2003.

[15] Horn JL. A rationale and test for the number of factors in factor analysis. Psychometrika. 1965; 32:179–85. 10.1007/BF02289447 14306381

[16] Laurie CC, Doheny KF, Mirel DB, Pugh EW, Bierut LJ, Bhangale T, et al Quality control and quality assurance in genotypic data for genome-wide association studies. Genet Epidemiol. 2010; 34:591–602. doi: 10.1002/gepi.20516 20718045 10.1002/gepi.20516 PMC3061487

[17] 1000 Genomes Project Consortium, Abecasis GR, Auton A, Brooks LD, DePristo MA, Durbin RM, et al An integrated map of genetic variation from 1,092 human genomes. Nature. 2012; 491:56–65. doi: 10.1038/nature11632 23128226 10.1038/nature11632 PMC3498066

[18] Delaneau O, Zagury JF, Marchini J. Improved whole-chromosome phasing for disease and population genetic studies. Nat Methods. 2013; 10:5–6. doi: 10.1038/nmeth.2307 23269371 10.1038/nmeth.2307

[19] Howie B, Donnelly P, Marchini J. A flexible and accurate genotype imputation method for the next gen- eration of genome-wide association studies. PLoS Genet. 2009; 5:e1000529 doi: 10.1371/journal.pgen.1000529 19543373 10.1371/journal.pgen.1000529 PMC2689936

[20] Howie B, Marchini J, Stephens M. Genotype imputation with thousands of genomes. G3. 2011; 1:457–470. doi: 10.1534/g3.111.001198 22384356 10.1534/g3.111.001198 PMC3276165

[21] Purcell S, Neale B, Todd-Brown K, Thomas L, Ferreira MA, Bender D, et al PLINK: a tool set for whole-genome association and population-based linkage analyses. Am J Hum Genet. 2007; 81:559–575. doi: 10.1086/519795 17701901 10.1086/519795 PMC1950838

[22] Smyth I, Du X, Taylor MS, Justice MJ, Beutler B, Jackson IJ. The extracellular matrix gene Frem1 is essential for the normal adhesion of the embryonic epidermis. Proc Natl Acad Sci U S A. 2004; 101:13560–5. doi: 10.1073/pnas.0402760101 15345741 10.1073/pnas.0402760101 PMC518794

[23] Alazami AM, Shaheen R, Alzahrani F, Snape K, Saggar A, Brinkmann B, et al FREM1 mutations cause bifid nose, renal agenesis, and anorectal malformations syndrome. Am J Hum Genet. 2009; 85:414–8. doi: 10.1016/j.ajhg.2009.08.010 19732862 10.1016/j.ajhg.2009.08.010 PMC2771533

[24] Vissers LE, Cox TC, Maga AM, Short KM, Wiradjaja F, Janssen IM, et al Heterozygous mutations of FREM1 are associated with an increased risk of isolated metopic craniosynostosis in humans and mice. PLoS Genet. 2011; 7:e1002278 doi: 10.1371/journal.pgen.1002278 21931569 10.1371/journal.pgen.1002278 PMC3169541

[25] Dixon MJ, Marazita ML, Beaty TH, Murray JC. Cleft lip and palate: understanding genetic and environmental influences. Nat Rev Genet. 2011; 12:167–78. doi: 10.1038/nrg2933 21331089 10.1038/nrg2933 PMC3086810

[26] Slavotinek AM, Baranzini SE, Schanze D, Labelle-Dumais C, Short KM, Chao R, et al Manitoba-oculo-tricho-anal (MOTA) syndrome is caused by mutations in FREM1. J Med Genet. 2011; 48:375–82. doi: 10.1136/jmg.2011.089631 21507892 10.1136/jmg.2011.089631 PMC4294942

[27] Kitada T, Asakawa S, Hattori N, Matsumine H, Yamamura Y, Minoshima S, et al Mutations in the parkin gene cause autosomal recessive juvenile parkinsonism. Nature. 1998; 392:605–8. doi: 10.1038/33416 9560156 10.1038/33416

[28] Williams FM, Bansal AT, van Meurs JB, Bell JT, Meulenbelt I, Suri P, et al Novel genetic variants associated with lumbar disc degeneration in northern Europeans: a meta-analysis of 4600 subjects. Ann Rheum Dis. 2013; 72:1141–8. doi: 10.1136/annrheumdis-2012-201551 22993228 10.1136/annrheumdis-2012-201551 PMC3686263

[29] Lu W, Cheng YC, Chen K, Wang H, Gerhard GS, Still CD, et al Evidence for several independent genetic variants affecting lipoprotein (a) cholesterol levels. Hum Mol Genet. 2015; 24:2390–400. doi: 10.1093/hmg/ddu731 25575512 10.1093/hmg/ddu731 PMC4380064

[30] Mira MT, Alcaïs A, Nguyen VT, Moraes MO, Di Flumeri C, Vu HT, et al Susceptibility to leprosy is associated with PARK2 and PACRG. Nature. 2004; 427:636–40. doi: 10.1038/nature02326 14737177 10.1038/nature02326

[31] Visel A, Thaller C, Eichele G. GenePaint.org: an atlas of gene expression patterns in the mouse embryo. Nucleic Acids Res. 2004; 32:D552–D6. doi: 10.1093/nar/gkh029 14681479 10.1093/nar/gkh029 PMC308763

[32] Rogner UC, Spyropoulos DD, Le Novère N, Changeux JP, Avner P. Control of neurulation by the nucleosome assembly protein-1-like 2. Nat Genet. 2000; 25:431–5. doi: 10.1038/78124 10932189 10.1038/78124

[33] Dickinson ME, Flenniken AM, Ji X, Teboul L, Wong MD, White JK, et al High-throughput discovery of novel developmental phenotypes. Nature. 2016; 537:508–514. doi: 10.1038/nature19356 27626380 10.1038/nature19356 PMC5295821

[34] Van Laarhoven PM, Neitzel LR, Quintana AM, Geiger EA, Zackai EH, Clouthier DE, et al Kabuki syndrome genes KMT2D and KDM6A: functional analyses demonstrate critical roles in craniofacial, heart and brain development. Hum Mol Genet. 2015; 24:4443–53. doi: 10.1093/hmg/ddv180 25972376 10.1093/hmg/ddv180 PMC4492403

