# Supplementary material for: Genome-wide association study of facial morphology reveals novel associations with FREM1 and PARK2
Source: PLoS One. 2017 Apr 25;12(4):e0176566. doi: 10.1371/journal.pone.0176566 (PMC5404842; doi:10.1371/journal.pone.0176566)
Supplement: S2 Table — (DOCX) [file pone.0176566.s002.docx]

**S2 Table:** Comparison of top associations in all participants and the subset of participants ages 16-40 years. Effect estimates are not meaningfully different and significance levels are similar.

|  |  | **All participants**  **N=2187** |  | **Ages 16-40**  **N=1719** |  |
| --- | --- | --- | --- | --- | --- |
| **Trait** | **Lead SNP** | **Beta (SE)** | **p** | **Beta (SE)** | **p** |
| Factor 3 | rs117438382 | 0.887 (0.161) | 3.76 × 10^-8^ | 0.895 (0.176) | 4.07 × 10^-7^ |
| Factor 7 | rs62031988 | 0.700(0.124) | 2.01 × 10^-8^ | 0.644 (0.140) | 4.30 × 10^-6^ |
| Factor 9 | rs9456748 | 0.165 (0.030) | 4.99 × 10^-8^ | 0.171 (0.034) | 5.86 × 10^-7^ |
| Factor 14 | rs11093404 | 0.175 (0.030) | 1.07 × 10^-8^ | 0.180 (0.035) | 3.77 × 10^-7^ |
| Factor 17 | rs72713618 | -0.592 (0.105) | 2.02 × 10^-8^ | -0.597 (0.114) | 1.83 × 10^-7^ |
| Factor 21 | rs113036800 | 0.691 (0.121) | 1.20 × 10^-8^ | 0.728 (0.127) | 1.22 × 10^-8^ |
| Factor 22 | rs138440928 | 0.650 (0.115) | 1.85 × 10^-8^ | 0.654 (0.126) | 2.36 × 10^-7^ |
