# Supplementary figures and images for: Genome-wide association study of facial morphology reveals novel associations with FREM1 and PARK2
Source: PLoS One. 2017 Apr 25;12(4):e0176566. doi: 10.1371/journal.pone.0176566 (PMC5404842; doi:10.1371/journal.pone.0176566)

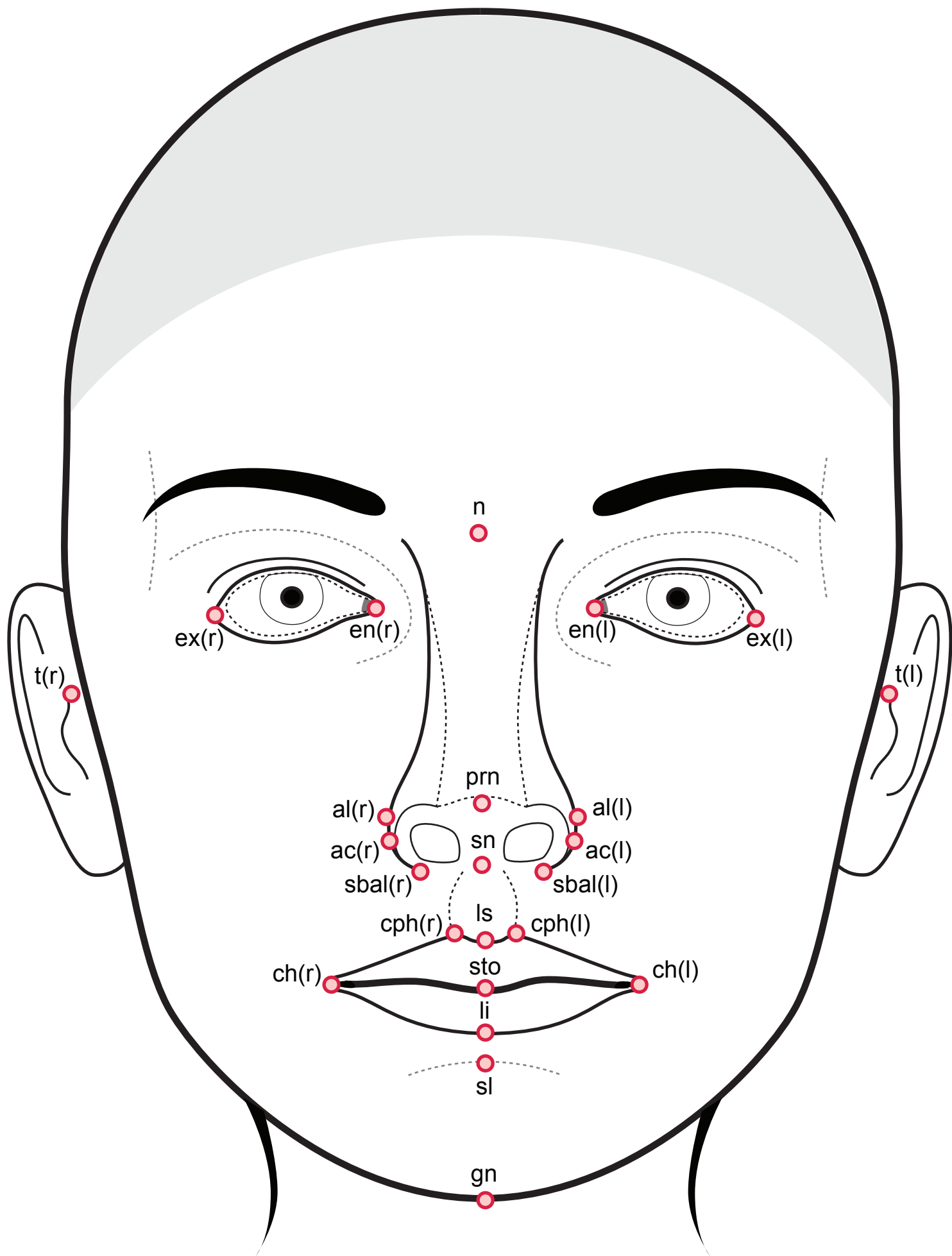

Supplement: S1 Fig — Landmarks are labeled as follows: n = nasion; prn = pronasale; sn = subnasale; ls = labiale superius; sto = stomion; li = labiale inferius; sl = sublabiale; gn = gnathion; en = endocanthion; ex = exocanthion; al = alare; ac = alar curvature point; sbal = subalare; cph = crista philtri; ch = chelion; and t = tragion. For bilateral landmarks, left and right indicated by _l and _r after the landmark abbreviation. (PDF) [file pone.0176566.s005.pdf]

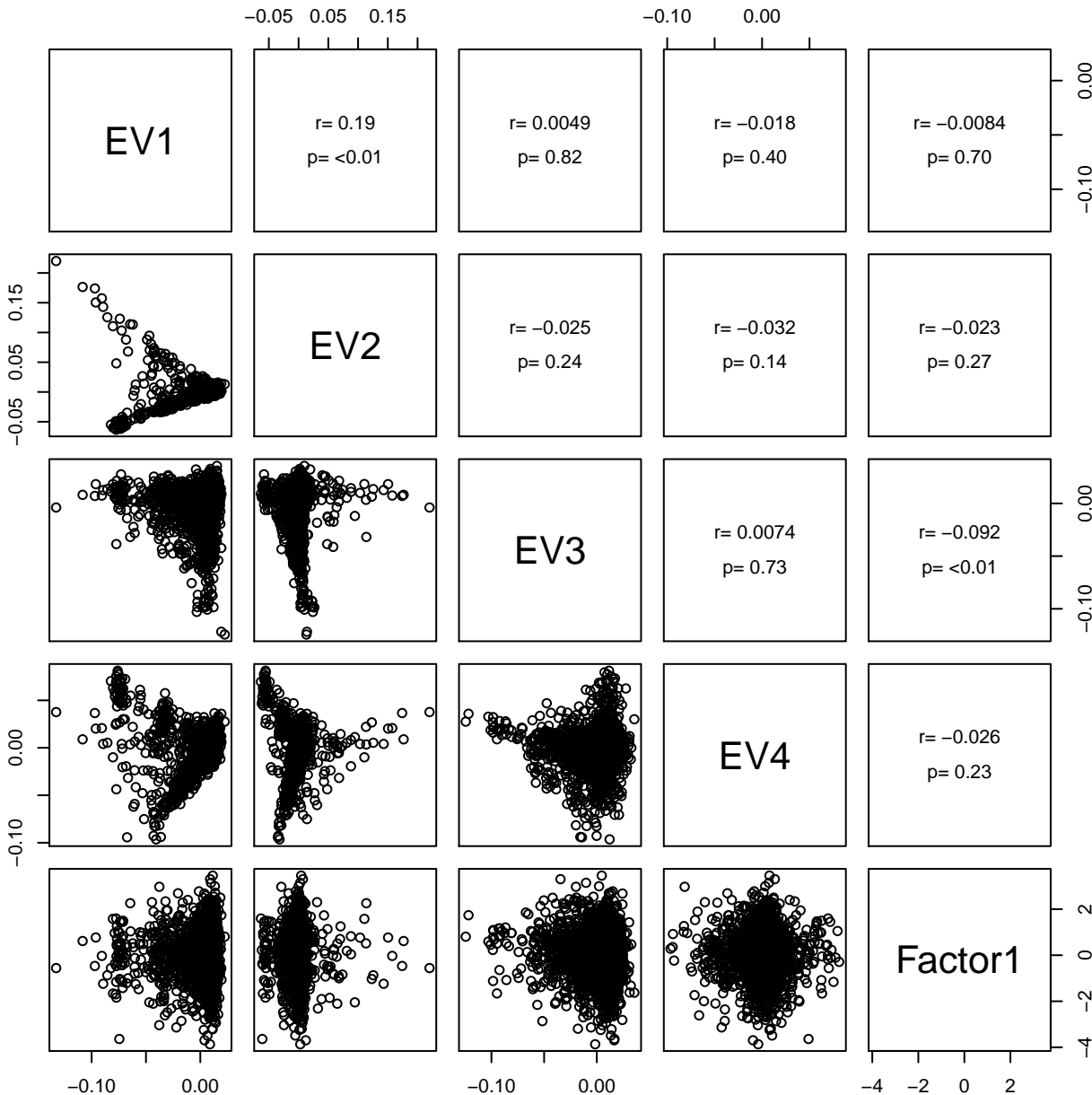

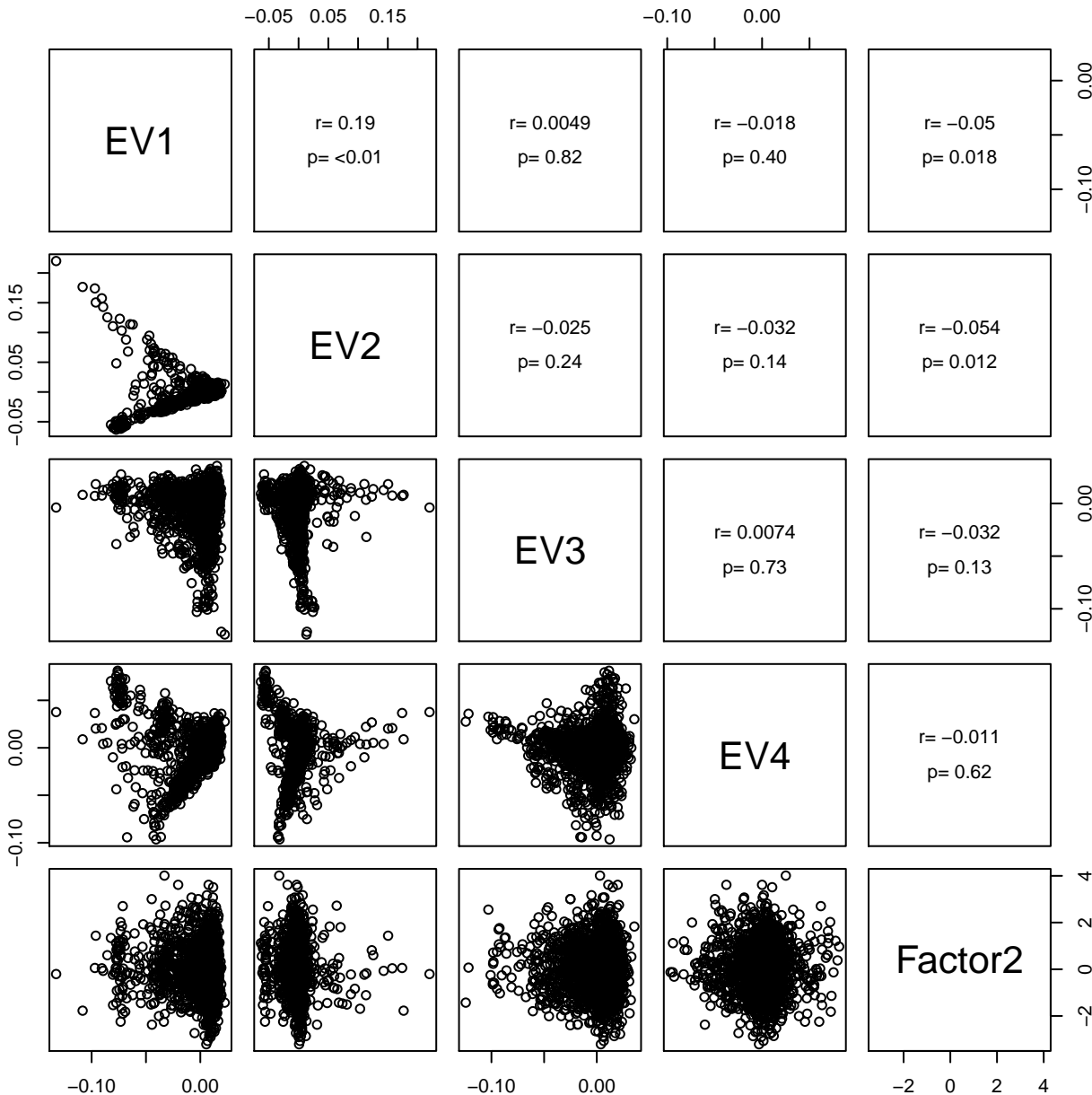

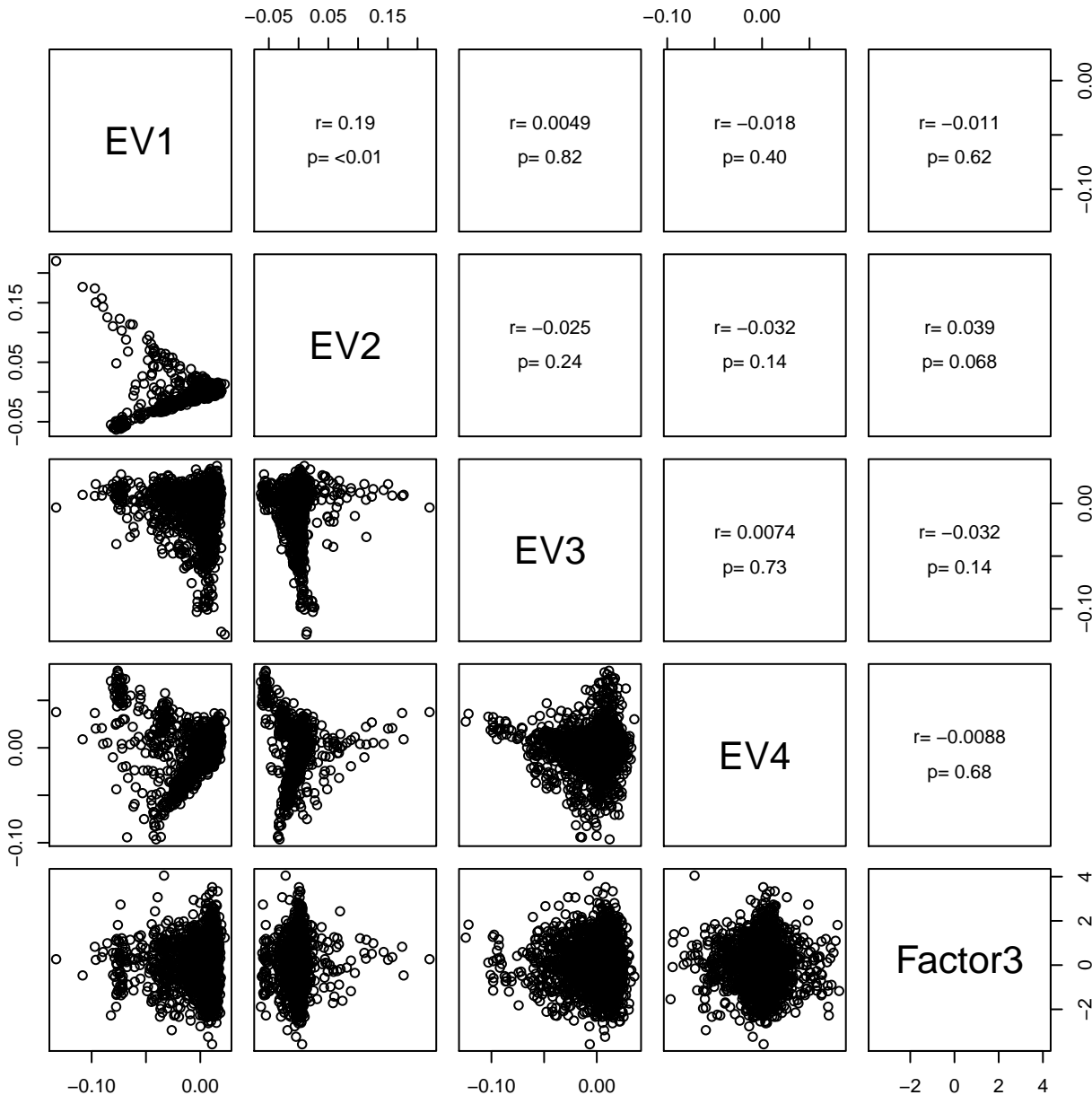

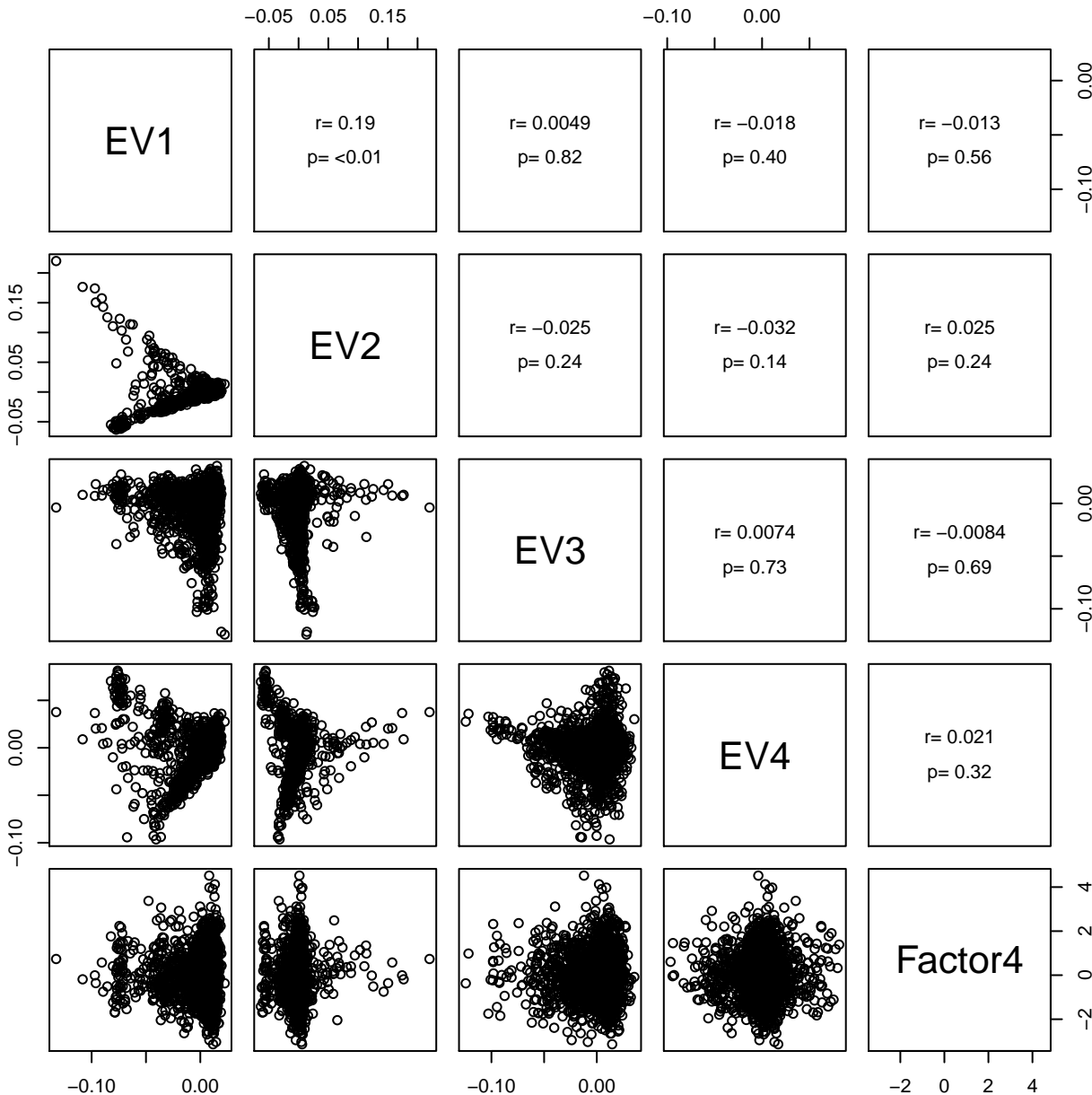

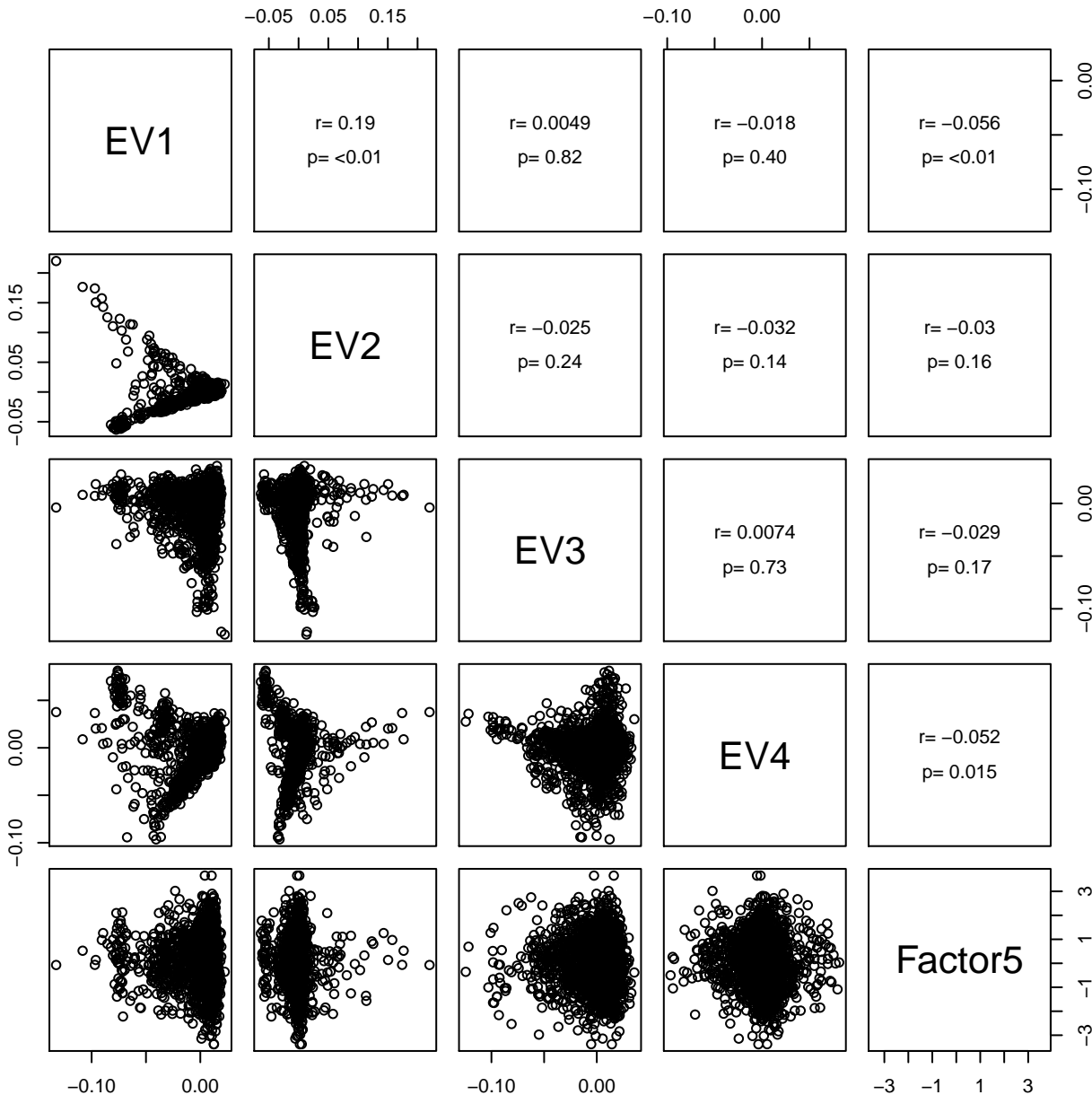

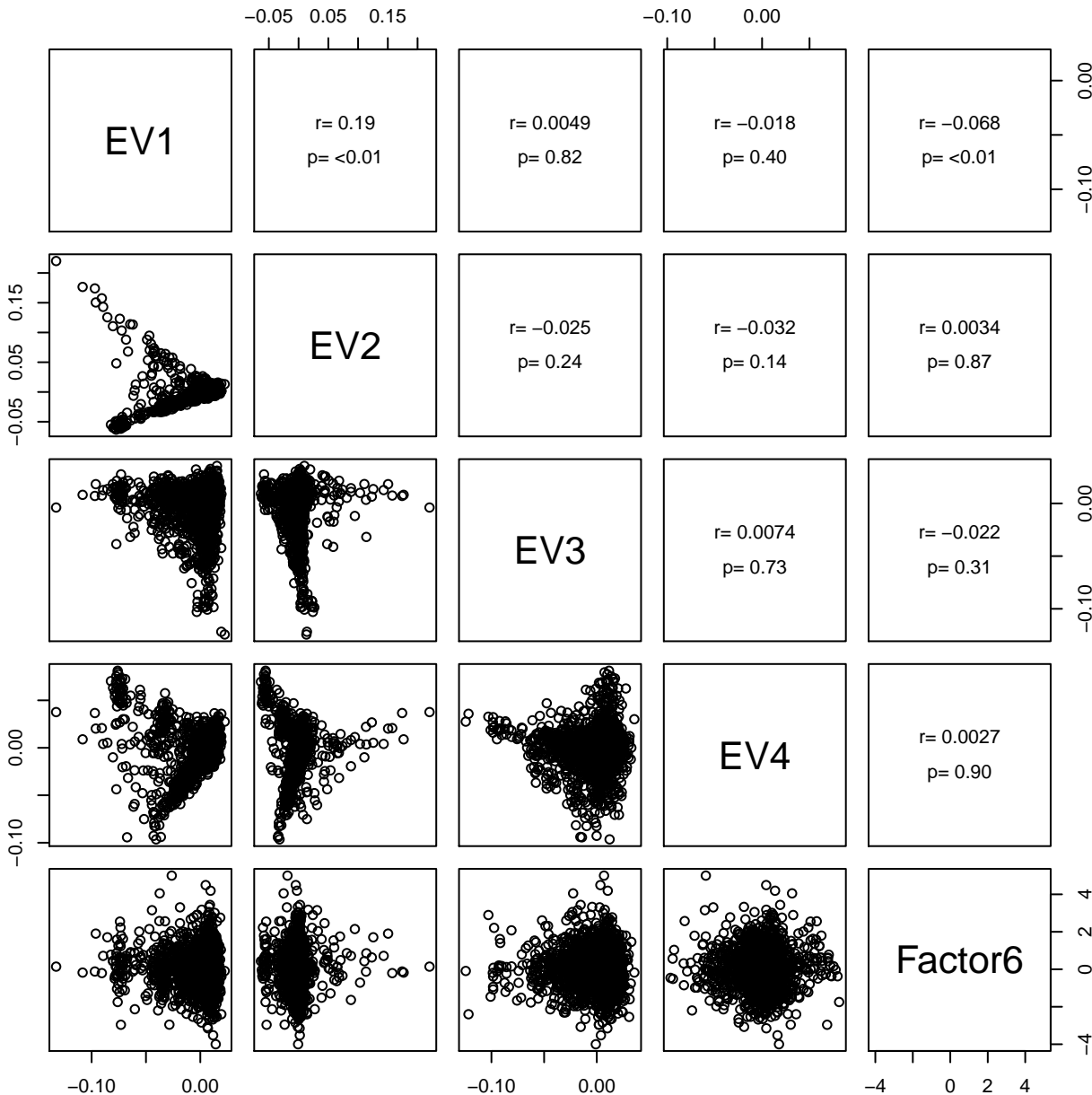

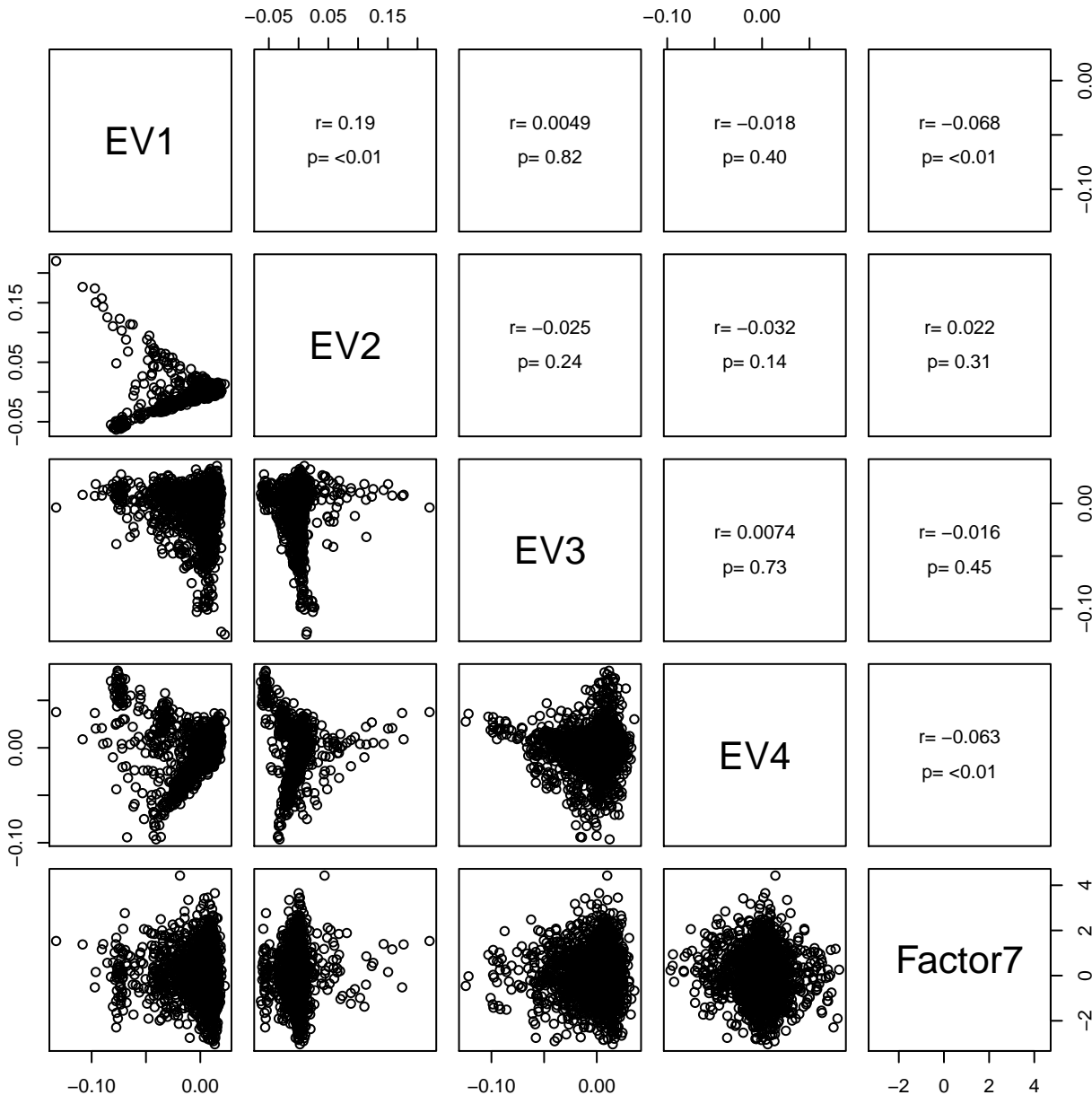

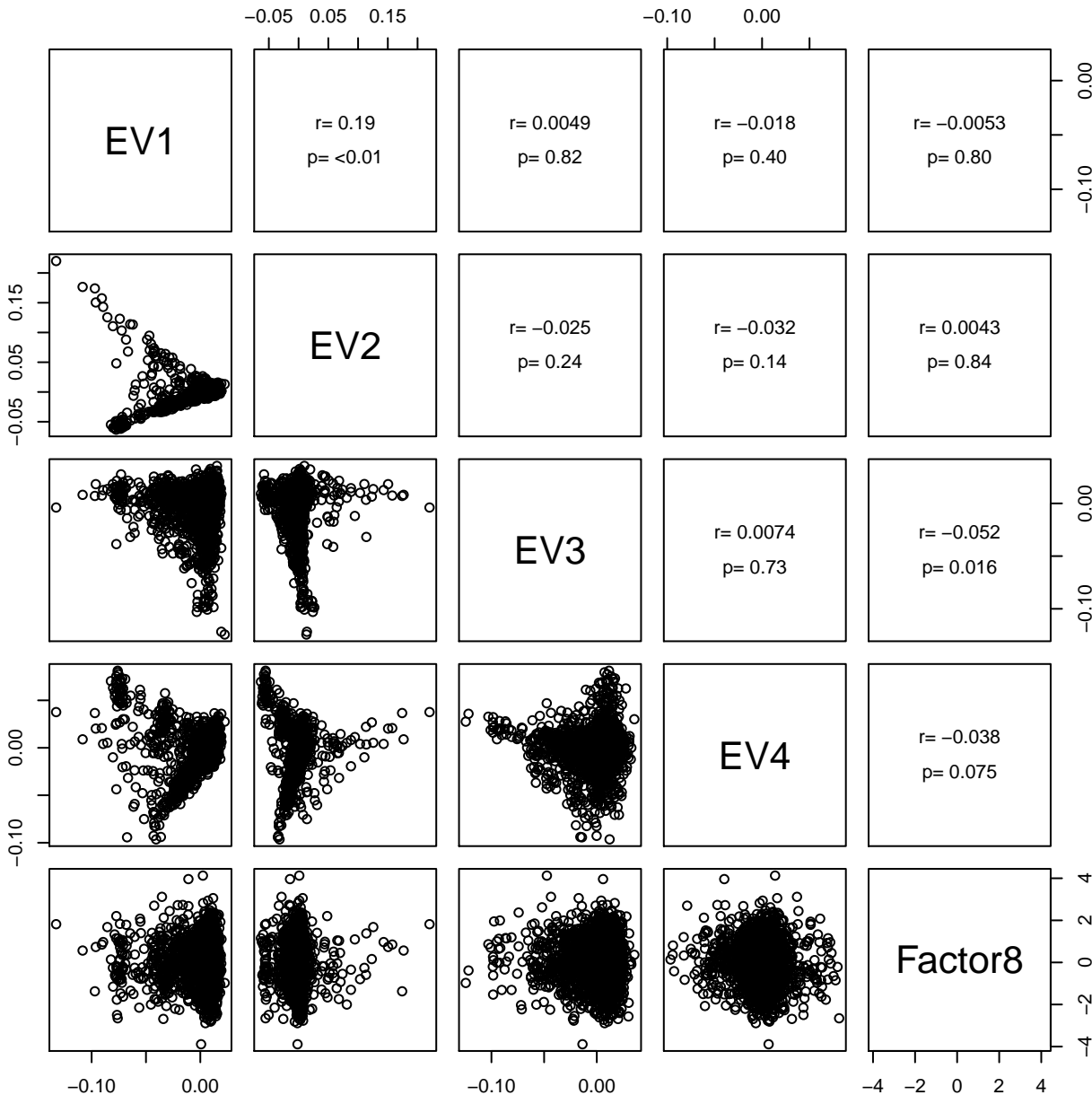

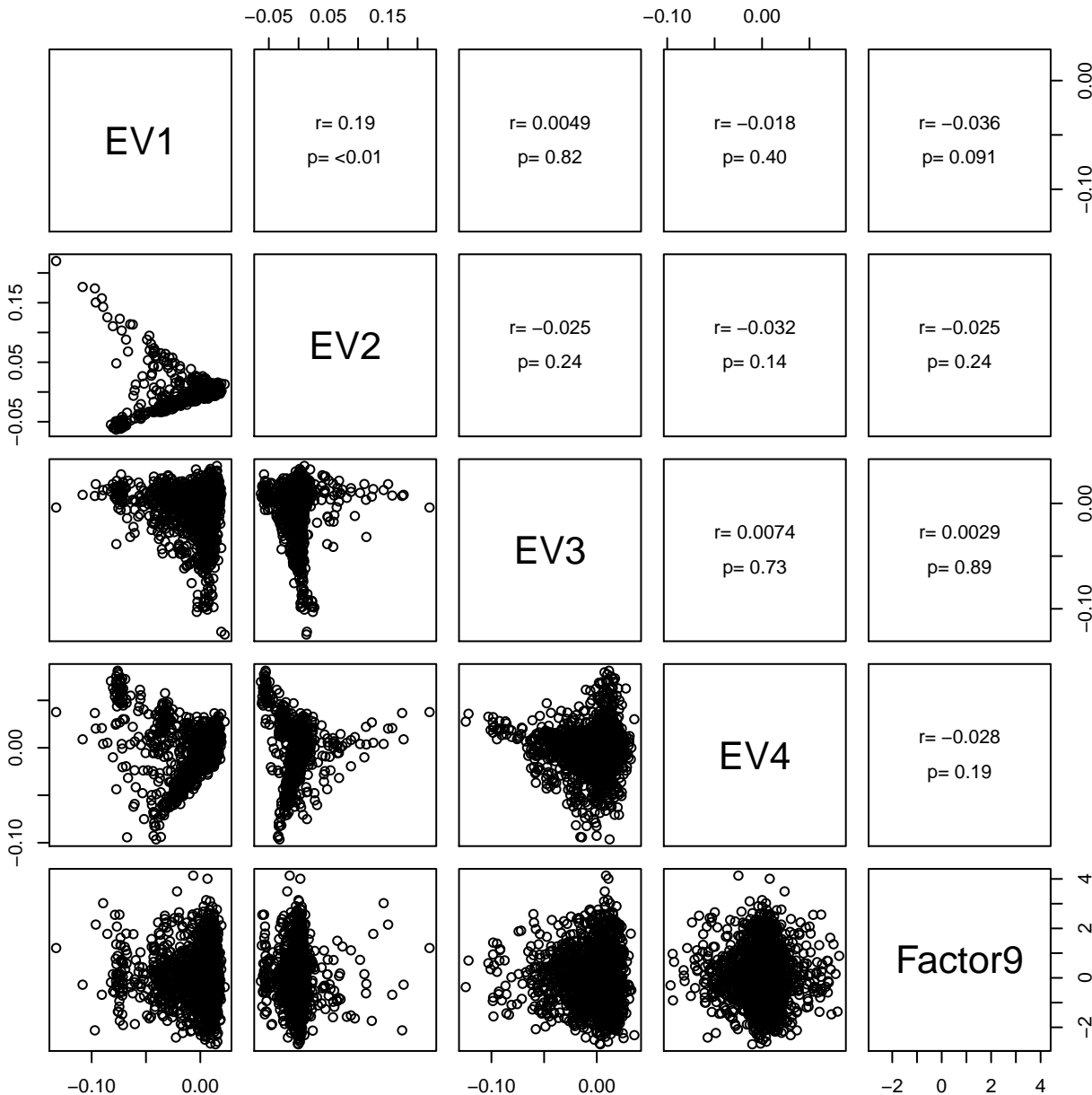

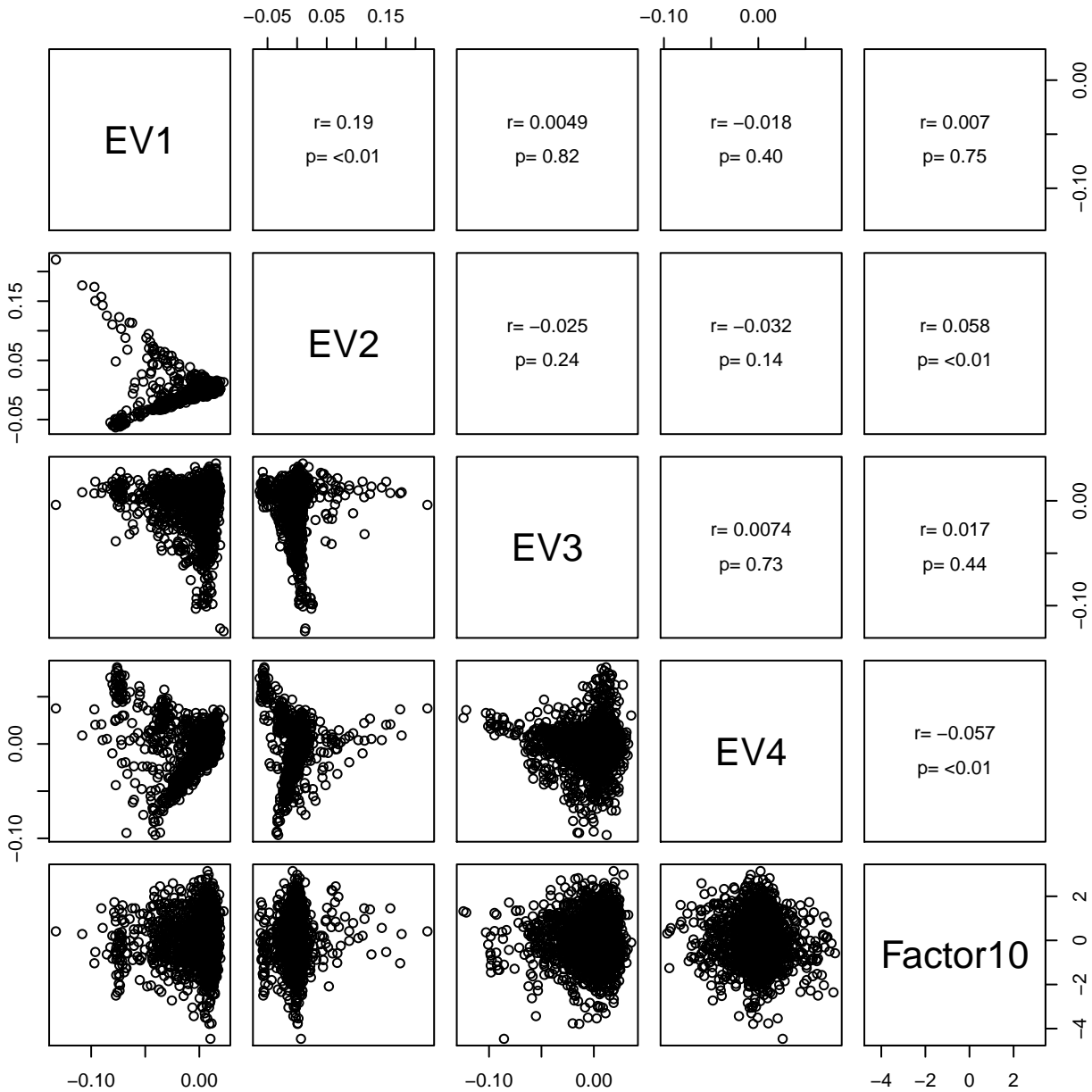

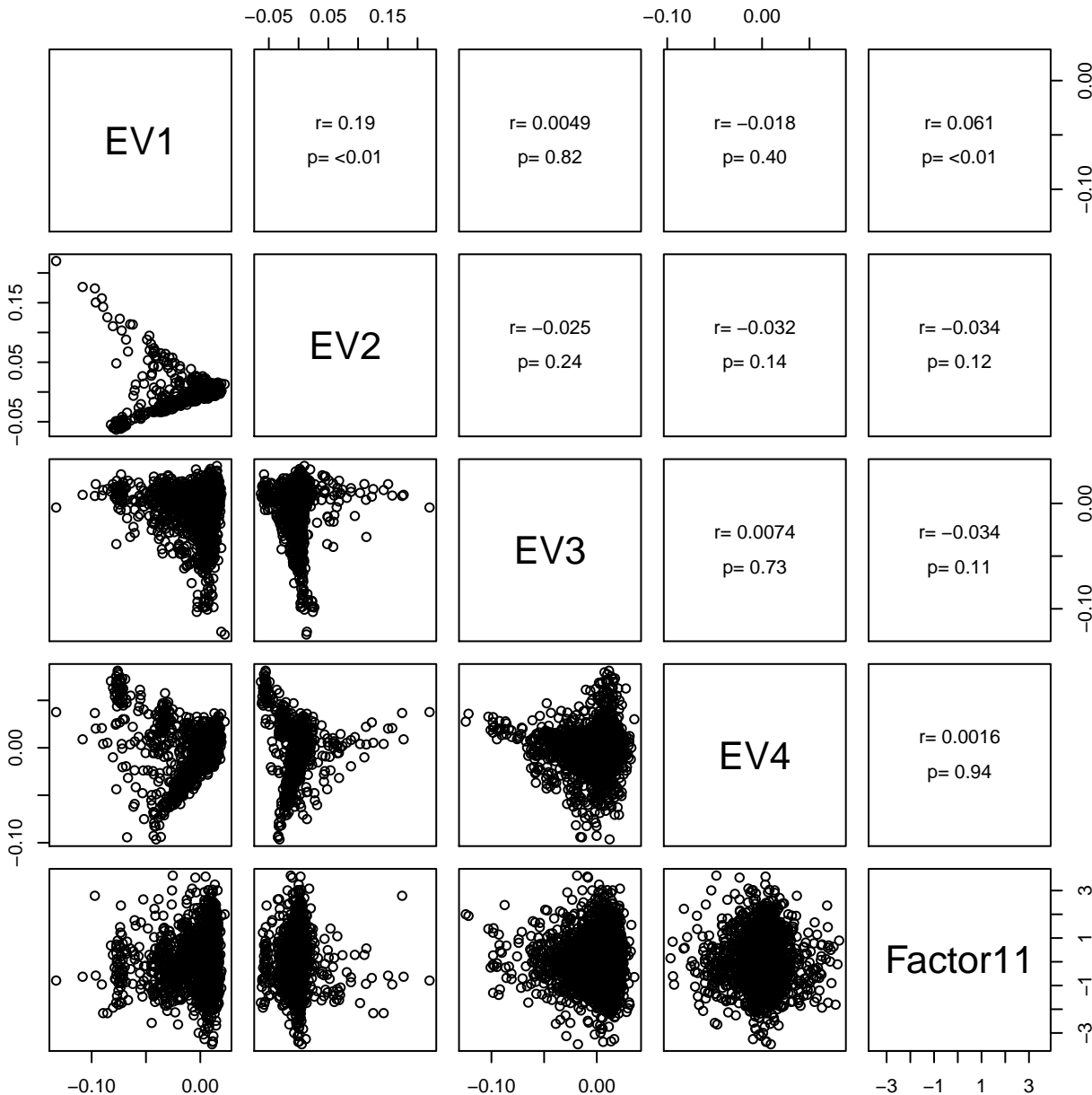

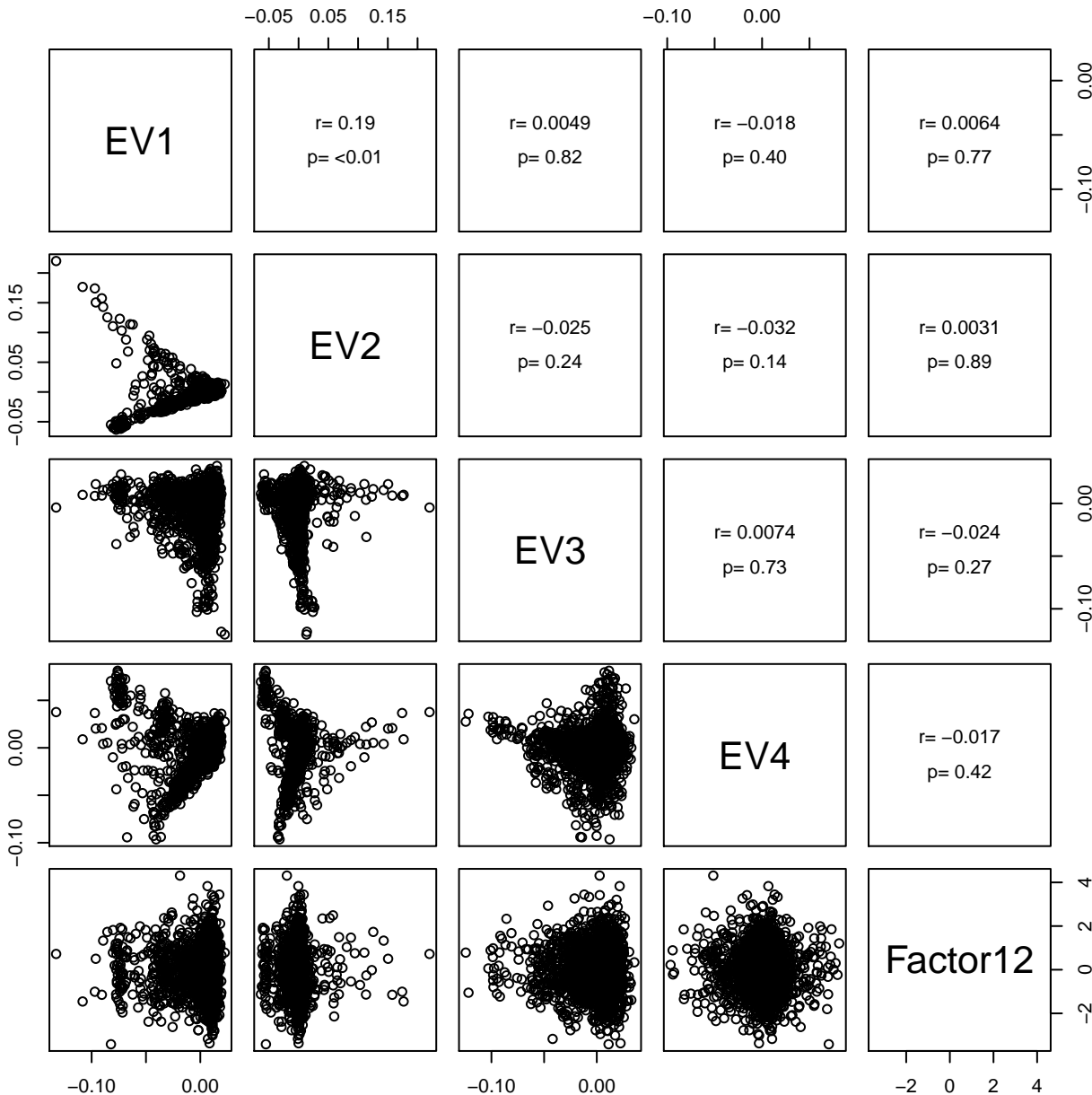

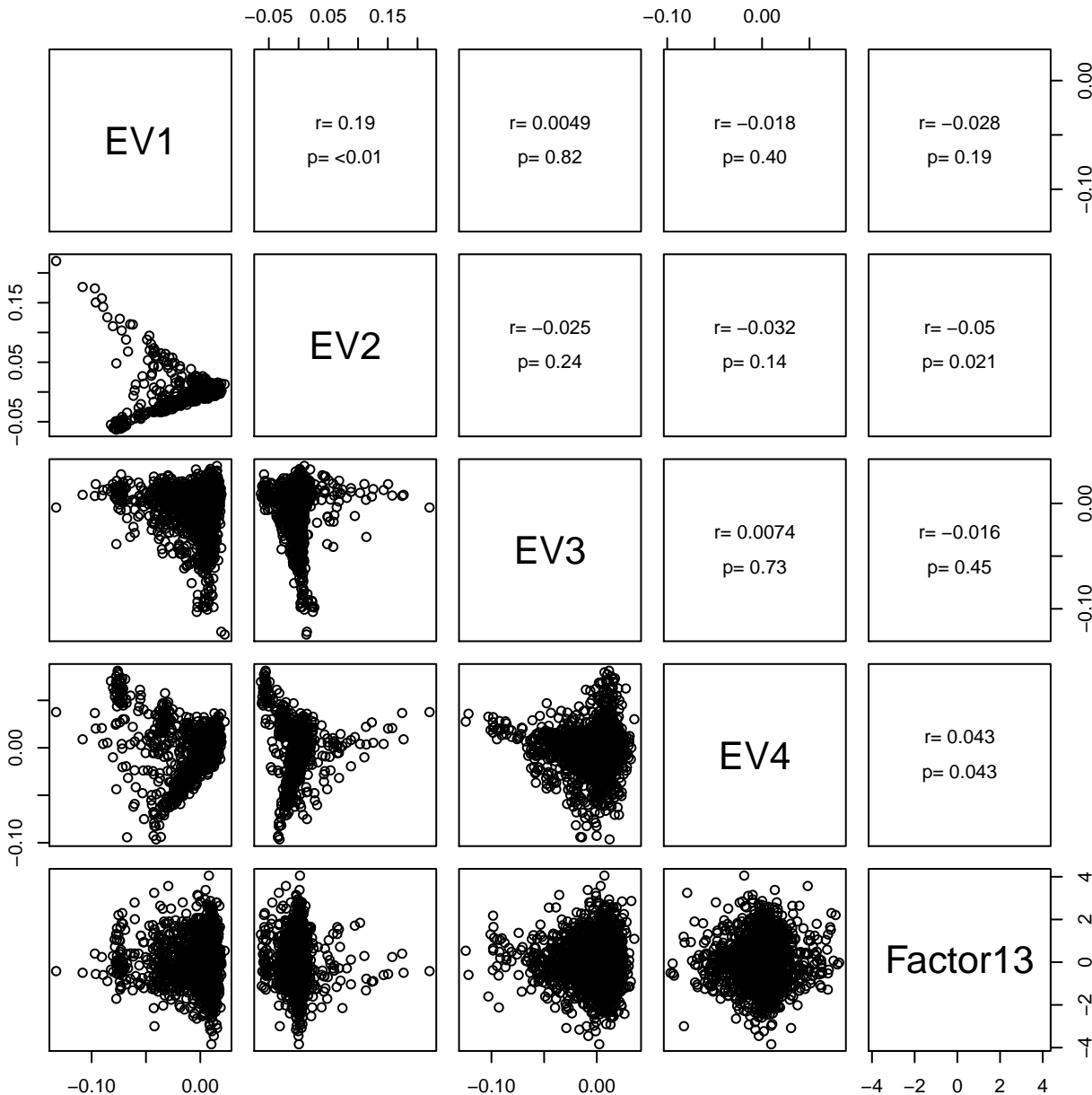

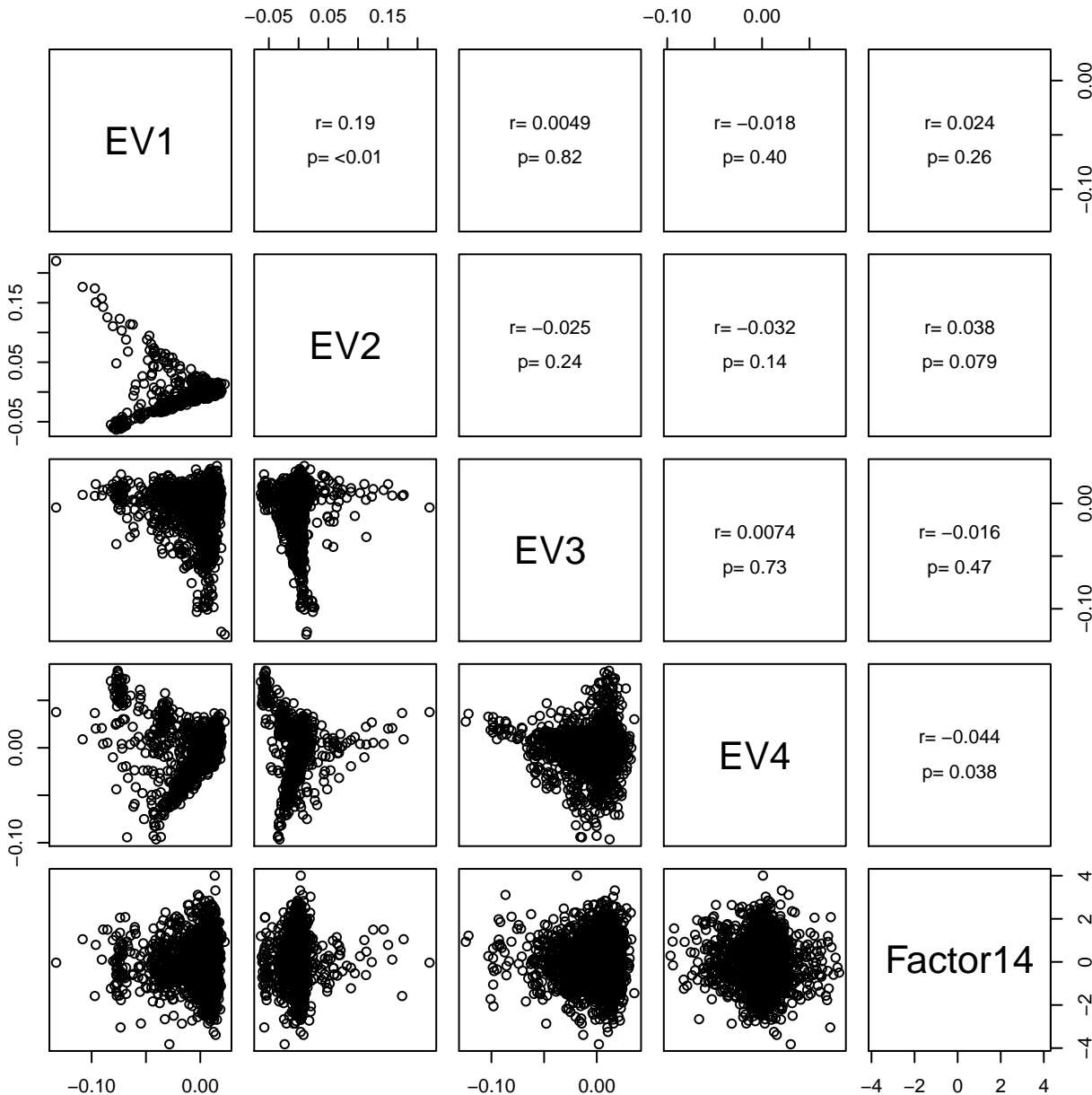

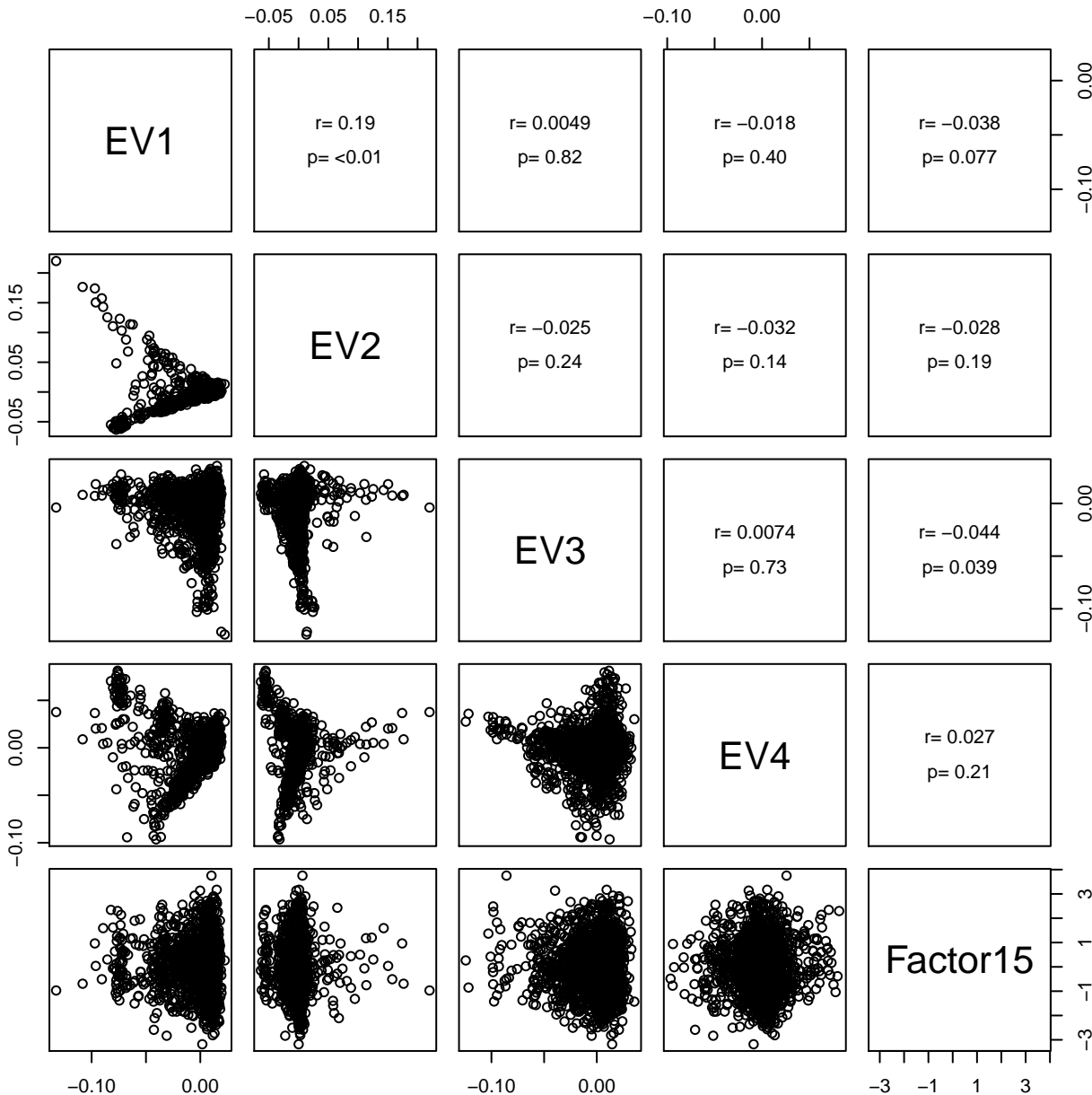

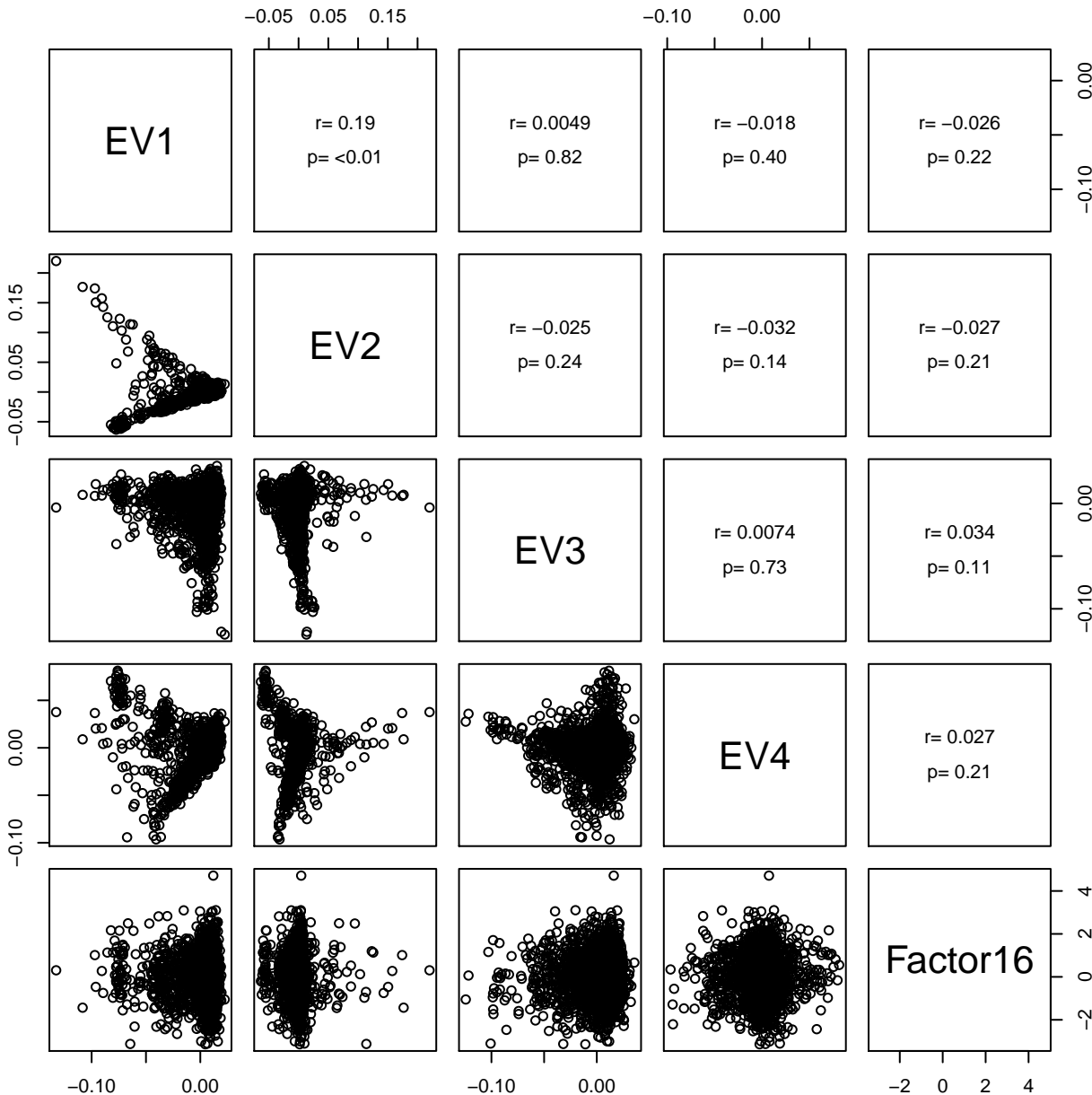

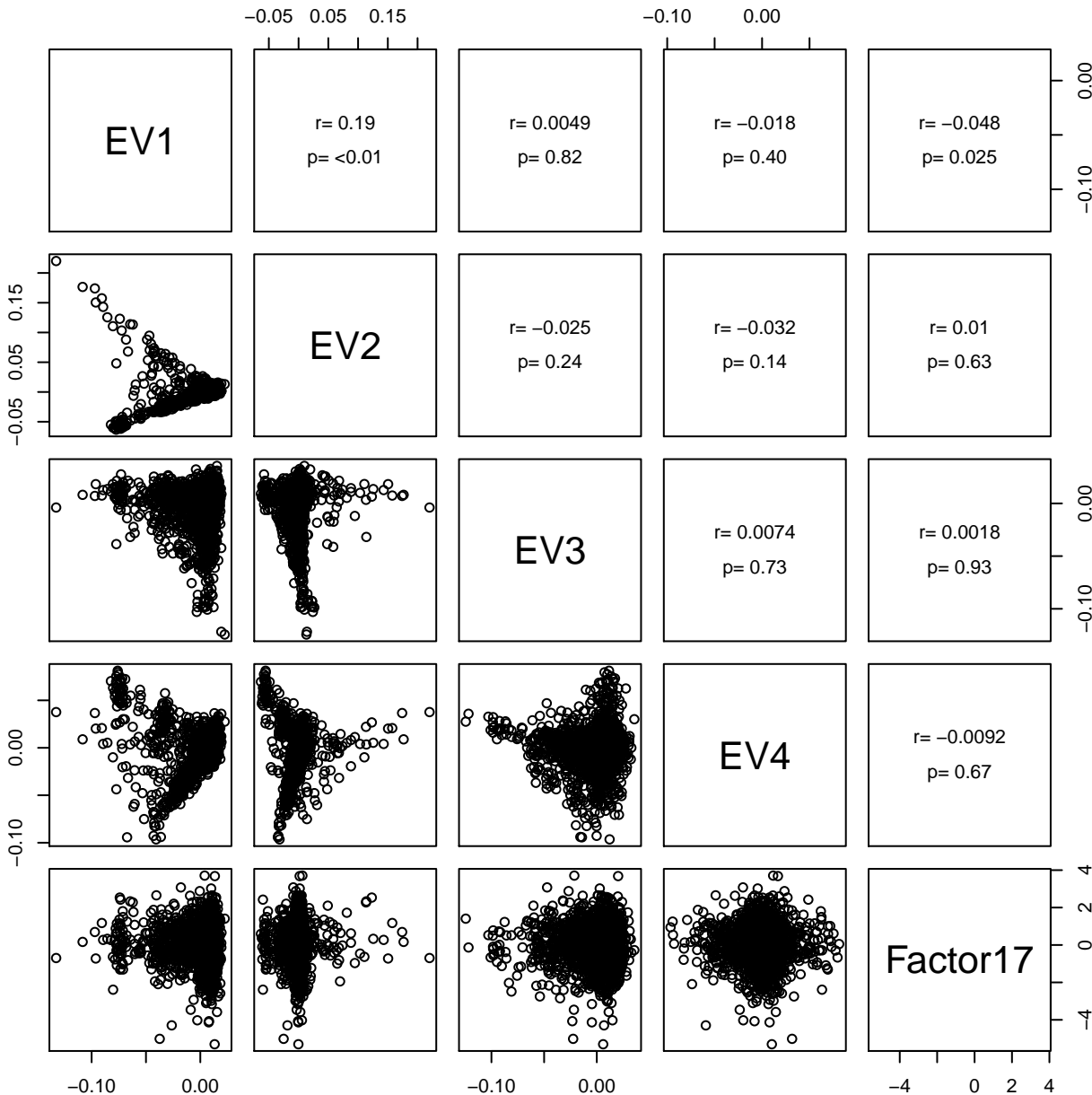

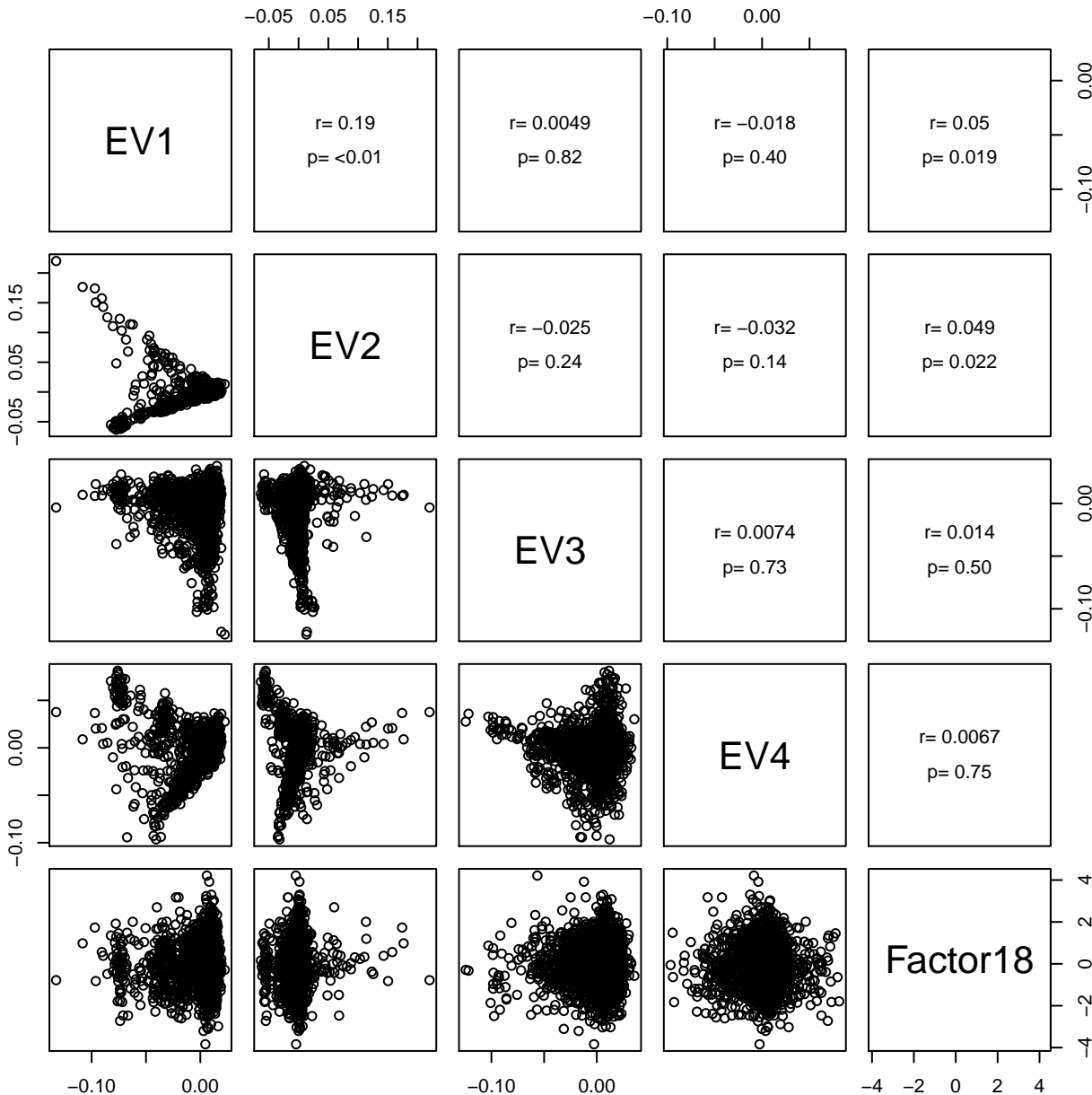

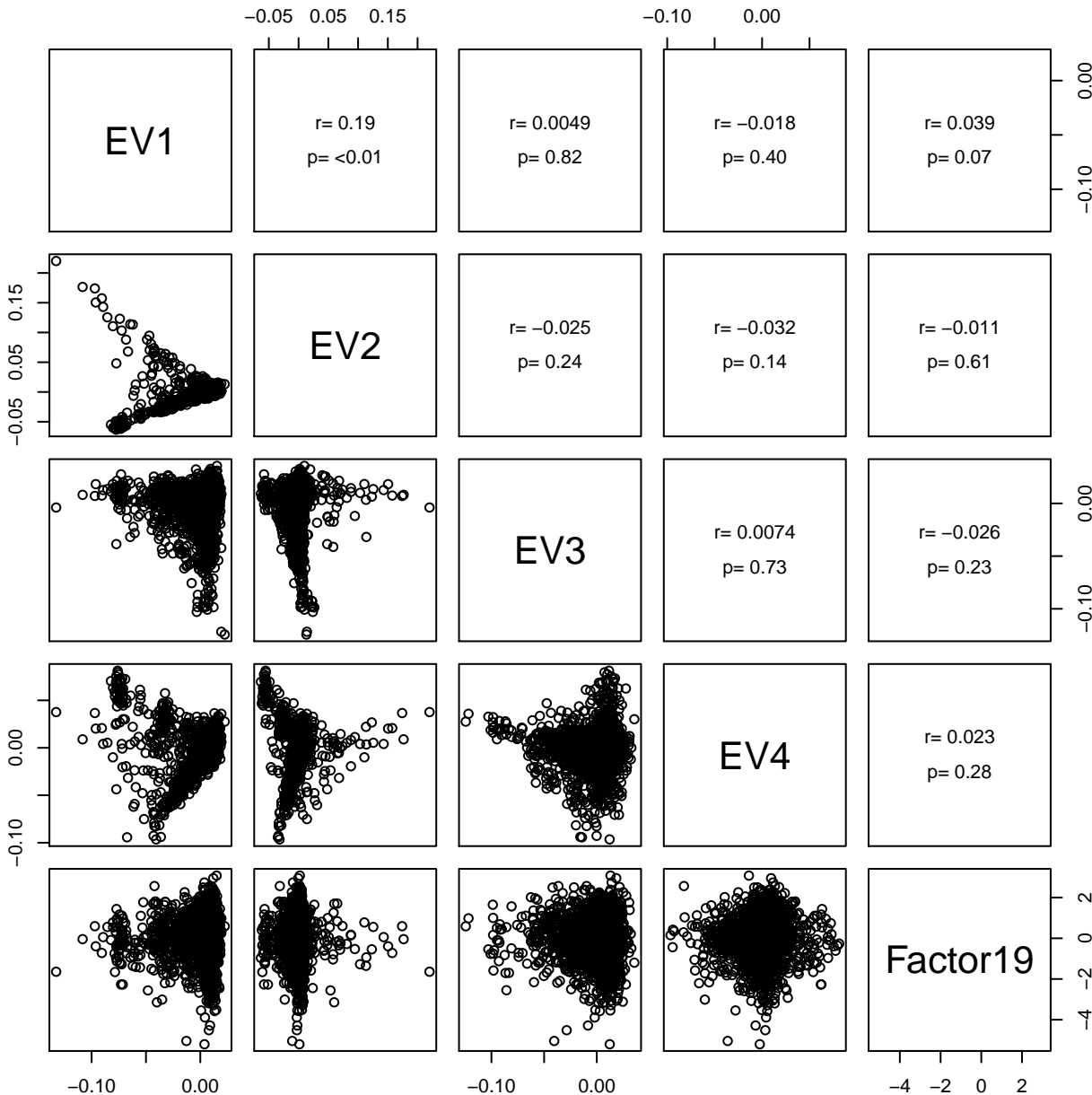

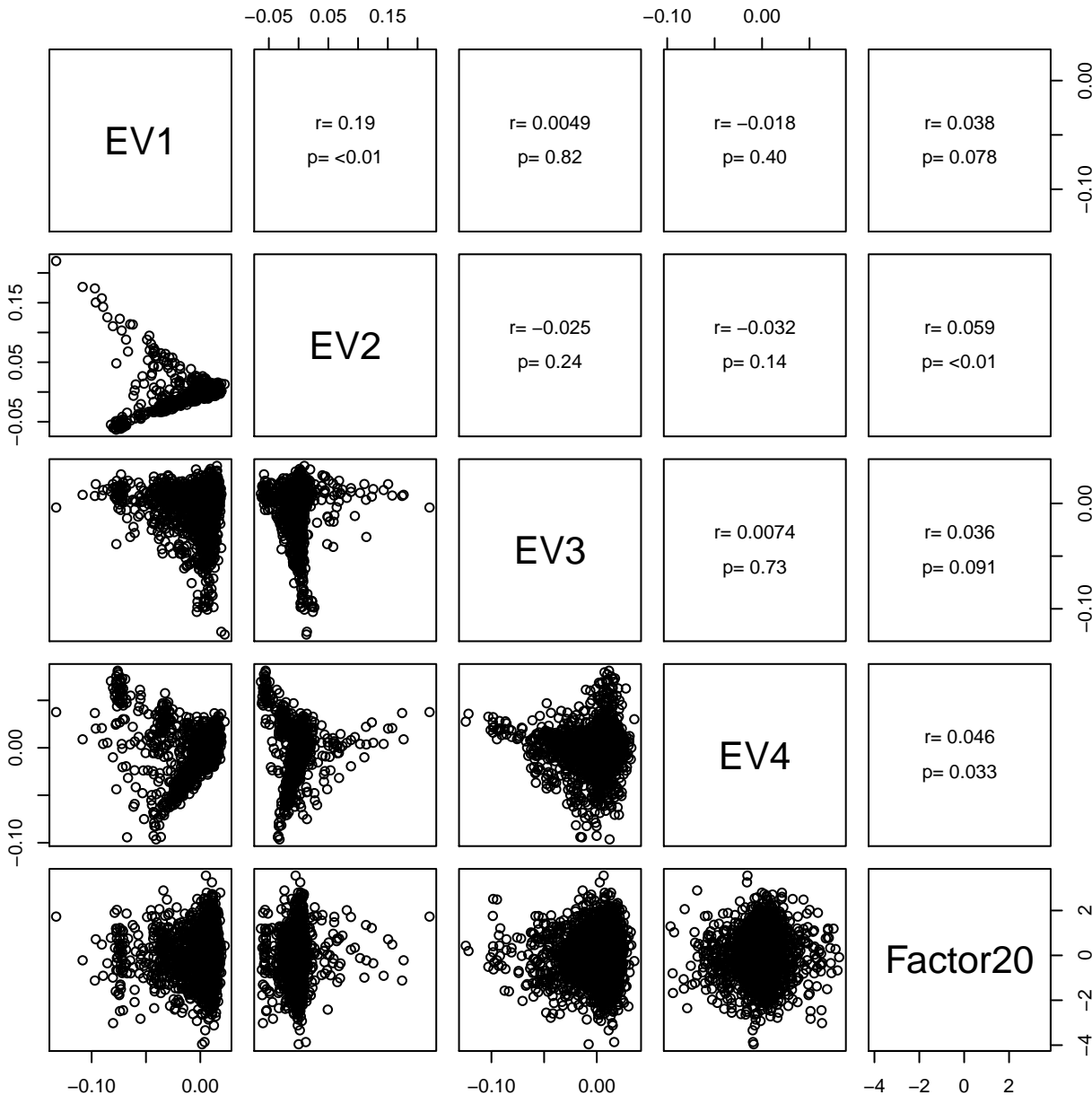

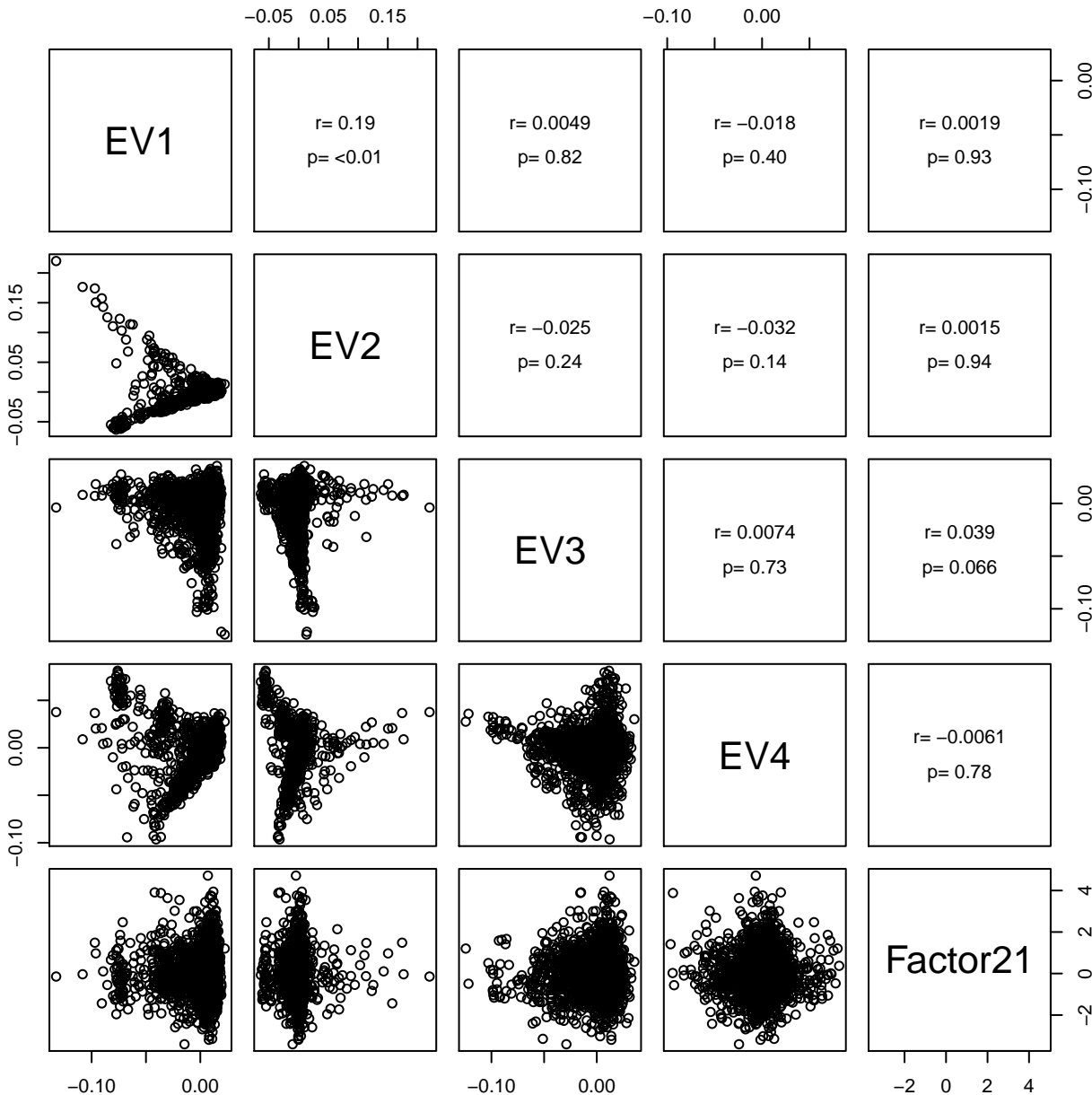

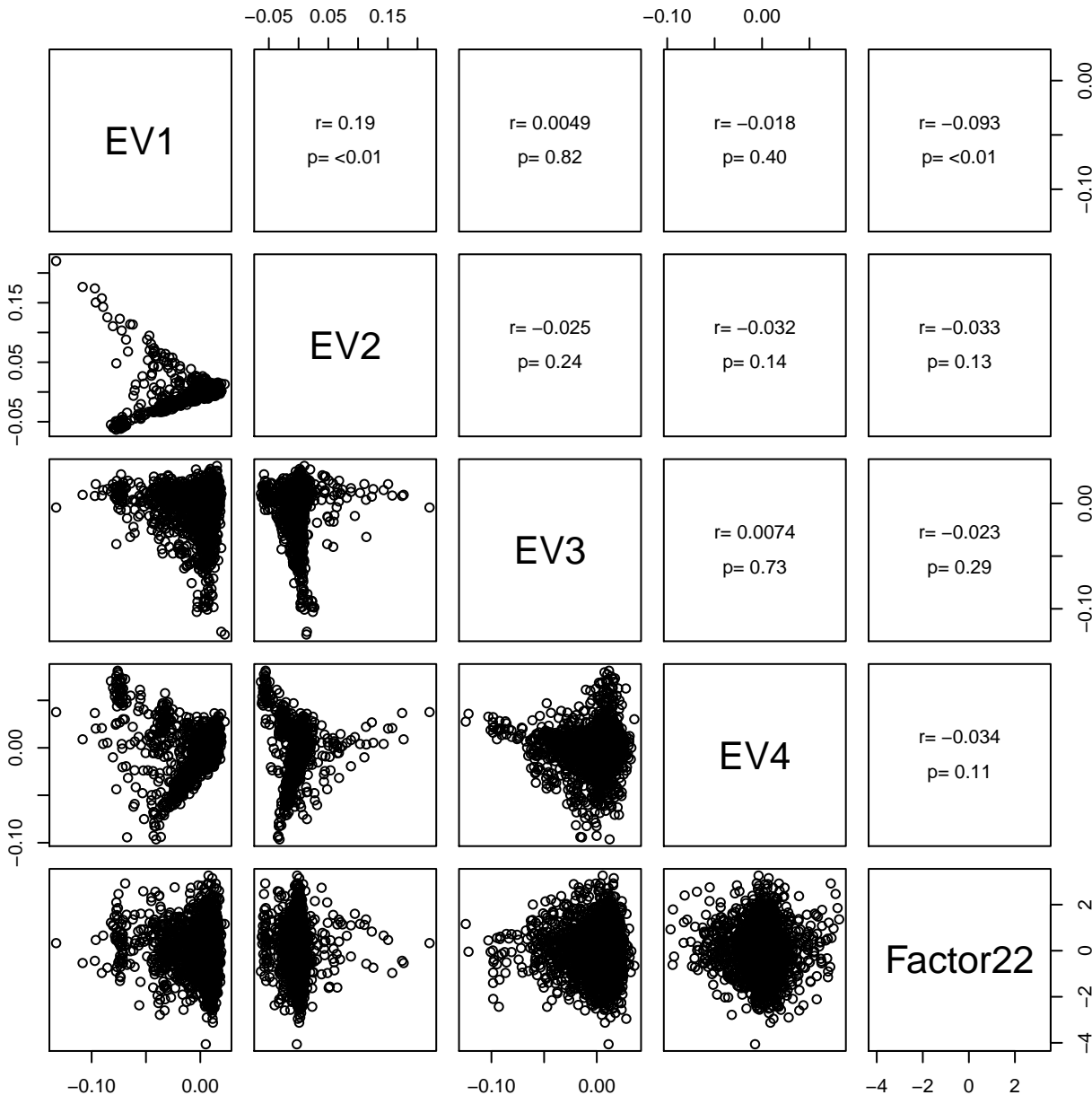

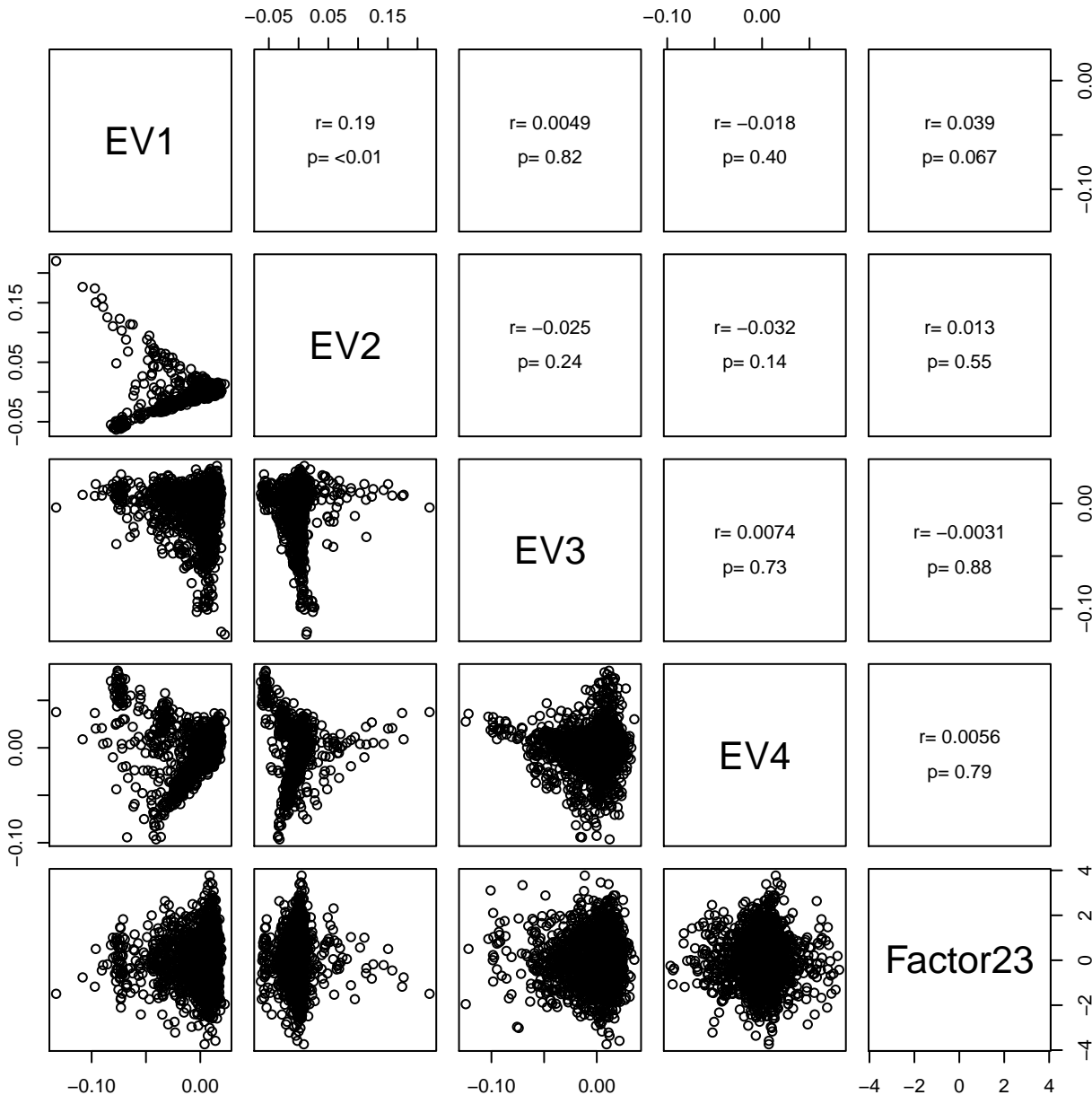

Supplement: S2 Fig — All pairwise combinations of Eigenvectors (EV; i.e., the values associated with each PC) and factors are depicted via scatterplots. Pearson correlation coefficient (r) and significance of the correlation (p) are indicated for each pair. (PDF) [file pone.0176566.s006.pdf]
