# Supplementary material for: Genome-wide association study of facial morphology reveals novel associations with FREM1 and PARK2
Source: PLoS One. 2017 Apr 25;12(4):e0176566. doi: 10.1371/journal.pone.0176566 (PMC5404842; doi:10.1371/journal.pone.0176566)
Supplement: S3 Fig — LocusZoom plots showing genome-wide significant associations observed for Factor 3 (A), Factor 21 (B), and Factor 22 (C). LocusZoom plots show the association (left y-axis; log10-transformed p-values) with each factor. Genotyped SNPs are depicted by stars and imputed SNPs are depicted by circles. Shading of the points represents the linkage disequilibrium (r2, based on the 1000 Genomes Project Europeans) between each SNP and the top SNP, indicated by purple shading. The blue overlay shows the recombination rate (right y-axis). Positions of genes are shown below the plot. (PDF) [file pone.0176566.s007.pdf]

A

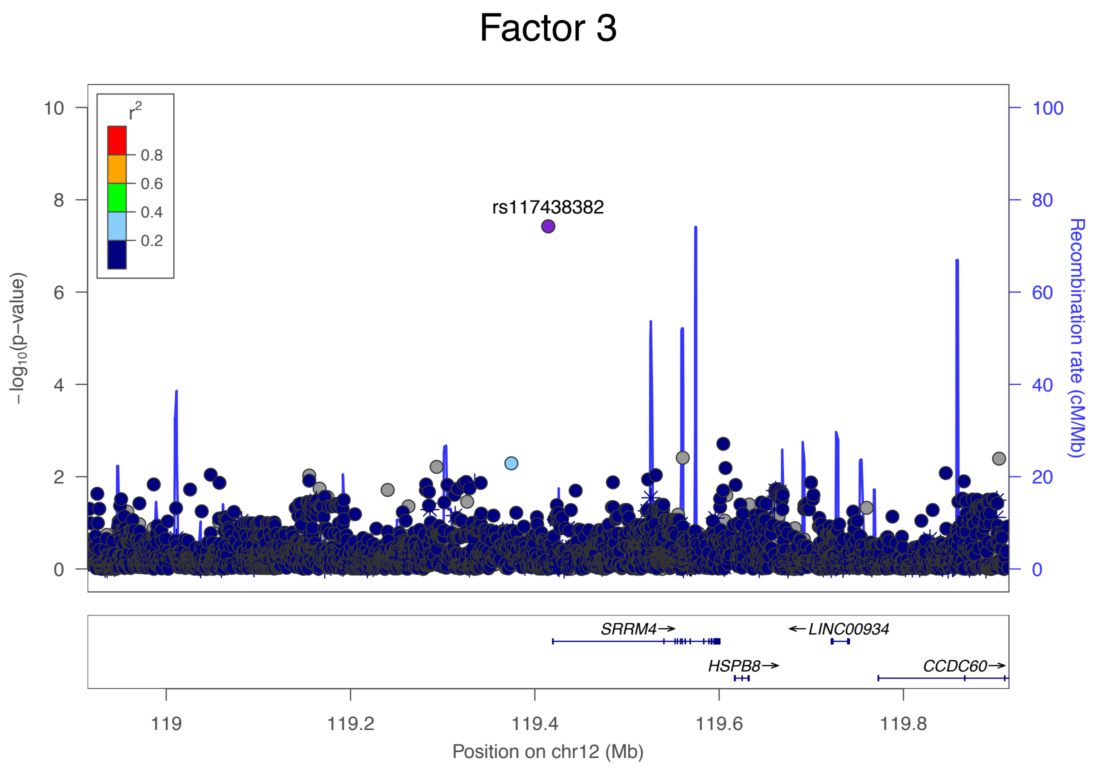
Factor 3
rs117438382
r2
0.8
0.6
0.4
0.2
−log10(p−value)
Recombination rate (cM/Mb)
SRRM4→
←LINC00934
HSPB8→
CCDC60→
Position on chr12 (Mb)
119
119.2
119.4
119.6
119.8

B

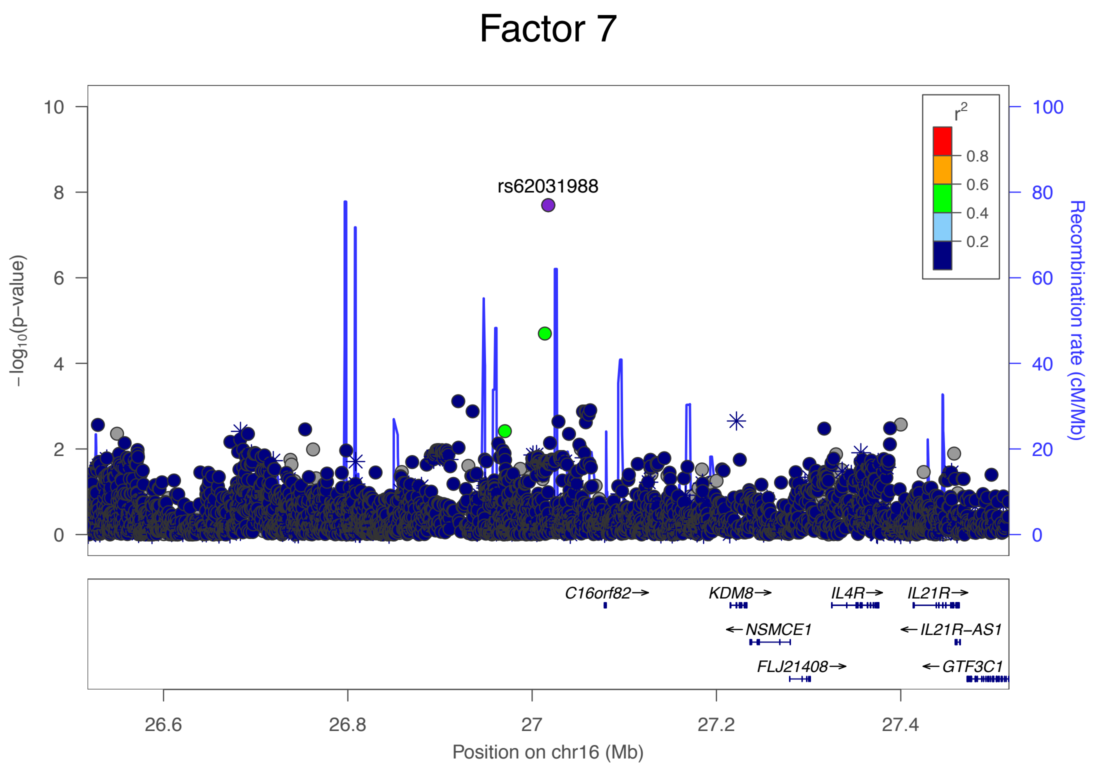
Factor 7
rs62031988
r2
0.8
0.6
0.4
0.2
−log10(p−value)
Recombination rate (cM/Mb)
C16orf82→
KDM8→
IL4R→
IL21R→
←NSMCE1
←IL21R−AS1
FLJ21408→
←GTF3C1
Position on chr16 (Mb)
26.6
26.8
27
27.2
27.4

C

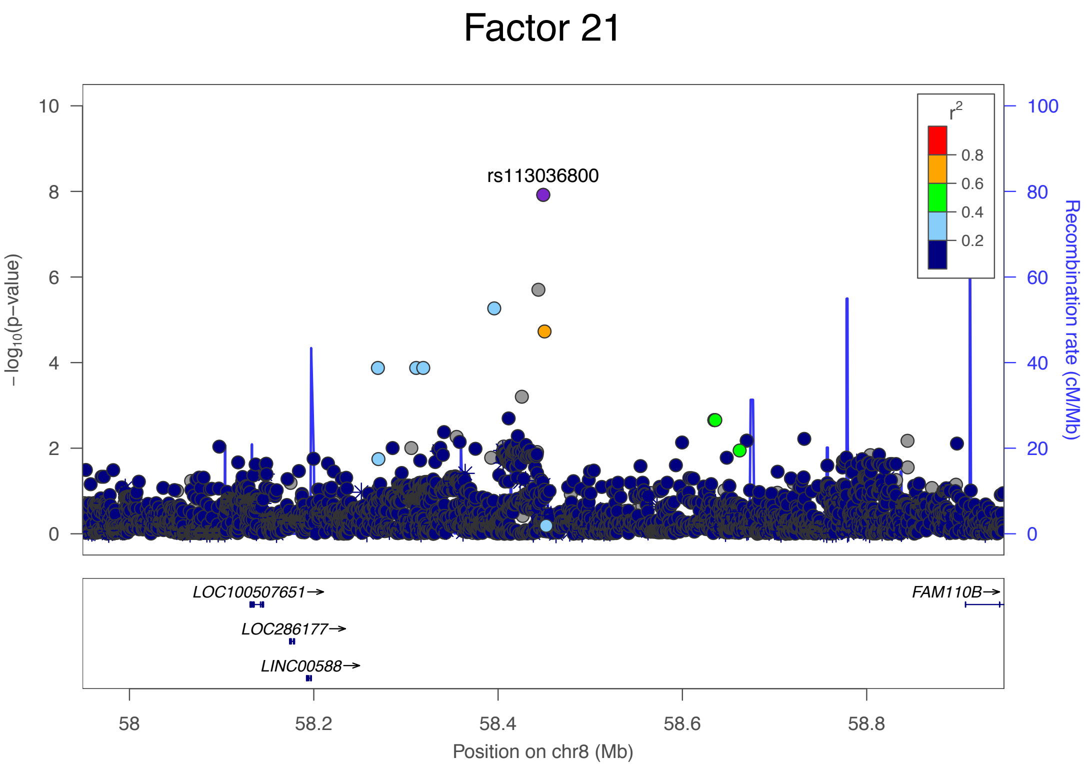

Factor 21
rs113036800
r2
0.8
0.6
0.4
0.2
10
8
6
4
2
0
100
80
60
40
20
0
−log10(p−value)
Recombination rate (cM/Mb)
LOC100507651→
LOC286177→
LINC00588→
FAM110B→
58
58.2
58.4
58.6
58.8
Position on chr8 (Mb)

D

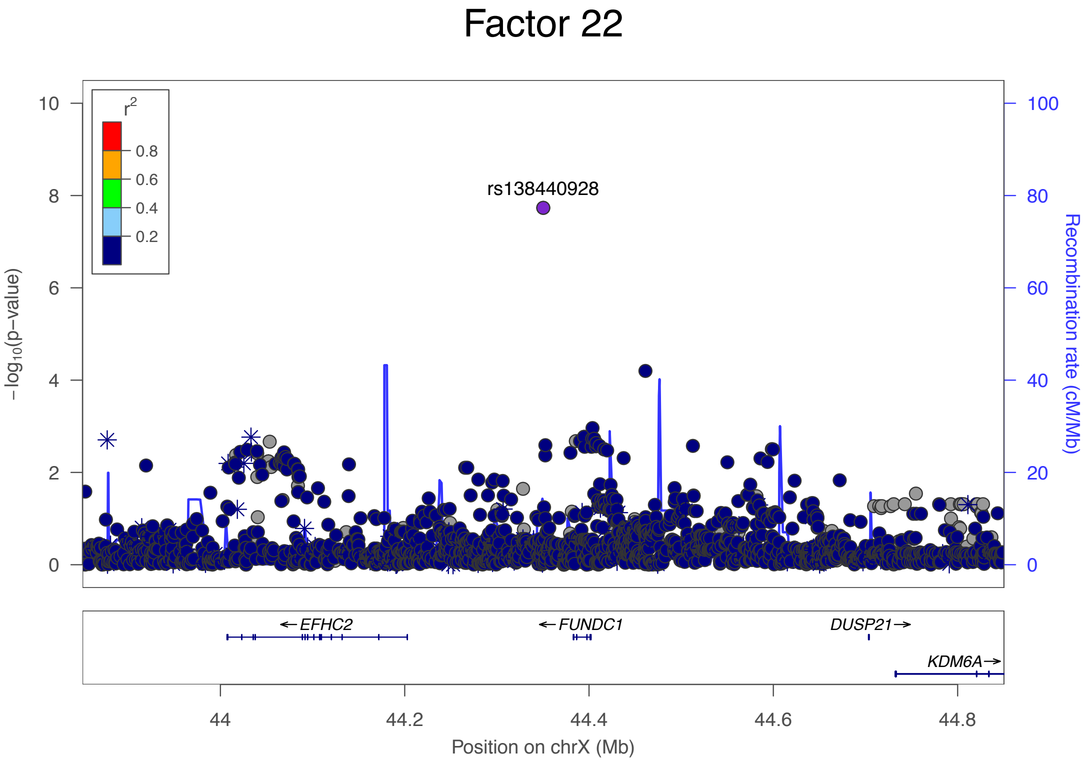

Factor 22
rs138440928
r2
0.8
0.6
0.4
0.2
10
8
6
4
2
0
100
80
60
40
20
0
−log10(p−value)
Recombination rate (cM/Mb)
←EFHC2
←FUNDC1
DUSP21→
KDM6A→
44
44.2
44.4
44.6
44.8
Position on chrX (Mb)

S2 Fig. LocusZoom plots showing genome-wide significant associations observed for Factor 3 (A), Factor 7 (B), Factor 21 (C), and Factor 22 (D). LocusZoom plots show the association (left y-axis; log10-transformed p-values) with each factor. Genotyped SNPs are depicted by stars and imputed SNPs are depicted by circles. Shading of the points represents the linkage disequilibrium (r2, based on the 1000 Genomes Project Europeans) between each SNP and the top SNP, indicated by purple shading. The blue overlay shows the recombination rate (right y-axis). Positions of genes are shown below the plot.
