# Supplementary material for: Genome-wide association study of facial morphology reveals novel associations with FREM1 and PARK2
Source: PLoS One. 2017 Apr 25;12(4):e0176566. doi: 10.1371/journal.pone.0176566 (PMC5404842; doi:10.1371/journal.pone.0176566)
Supplement: S4 Fig — Chromosomes are arranged in order along the x-axis. The y-axis shows the log base 10 p-value. Lines for p-value thresholds set at 5 x 10^−8^ for genome-wide significance and 5 x 10^−6^ for suggestive significance. (PDF) [file pone.0176566.s008.pdf]

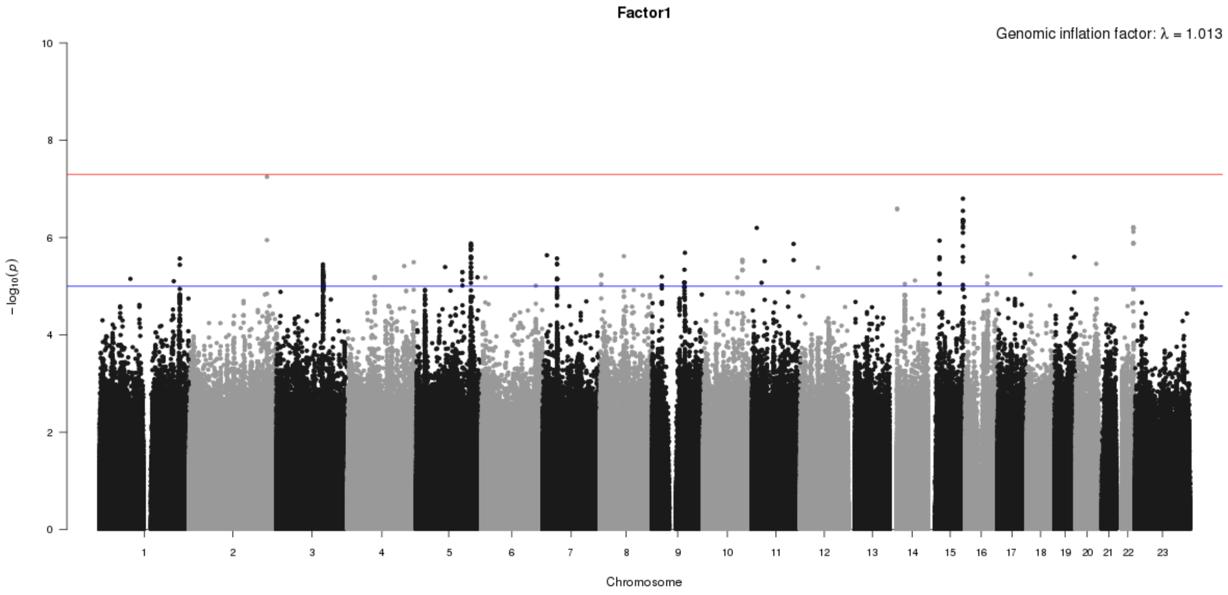
Factor1
Genomic inflation factor: λ = 1.013
−log10(p)
Chromosome
1 2 3 4 5 6 7 8 9 10 11 12 13 14 15 16 17 18 19 20 21 22 23

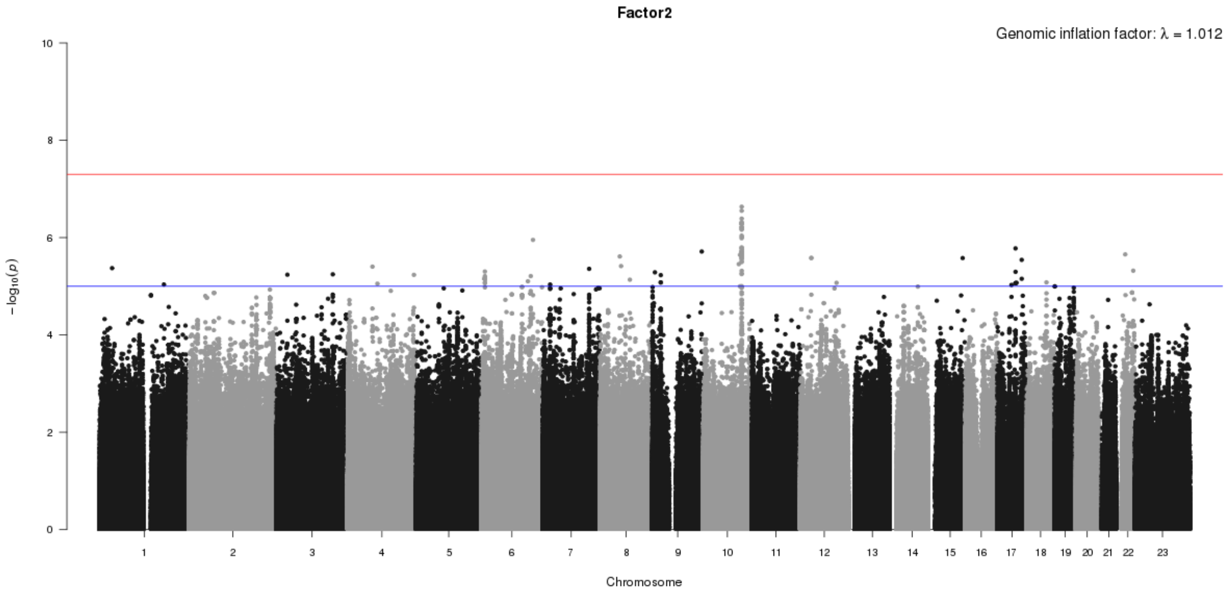
Factor2
Genomic inflation factor: λ = 1.012
−log10(p)
Chromosome
1 2 3 4 5 6 7 8 9 10 11 12 13 14 15 16 17 18 19 20 21 22 23

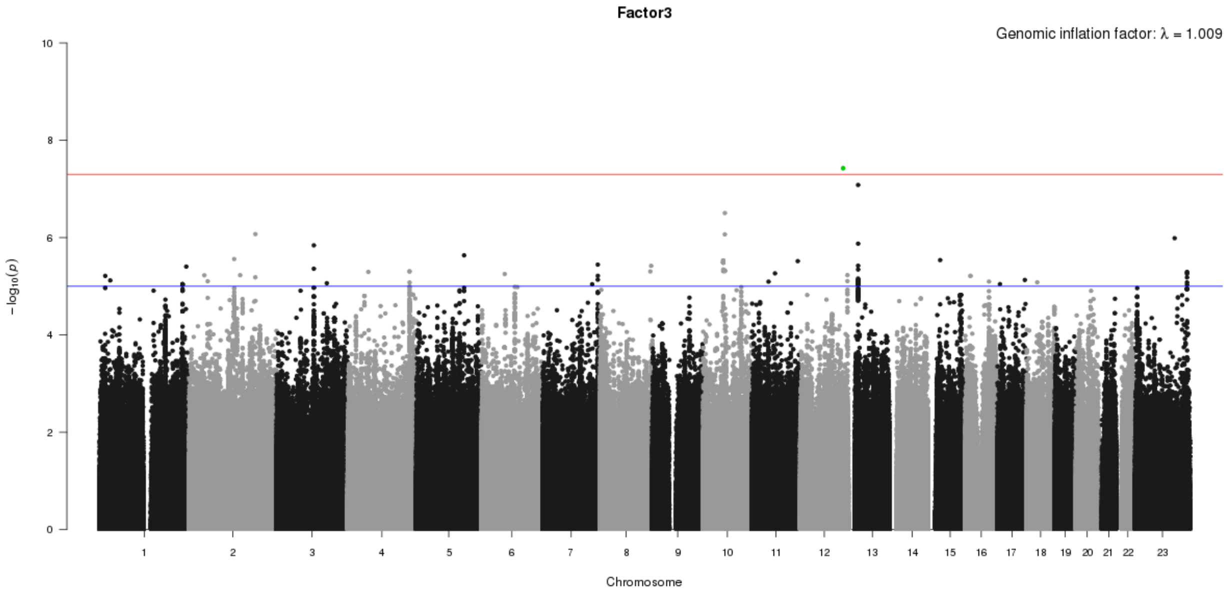
Factor3
Genomic inflation factor: λ = 1.009
−log10(p)
Chromosome
1 2 3 4 5 6 7 8 9 10 11 12 13 14 15 16 17 18 19 20 21 22 23

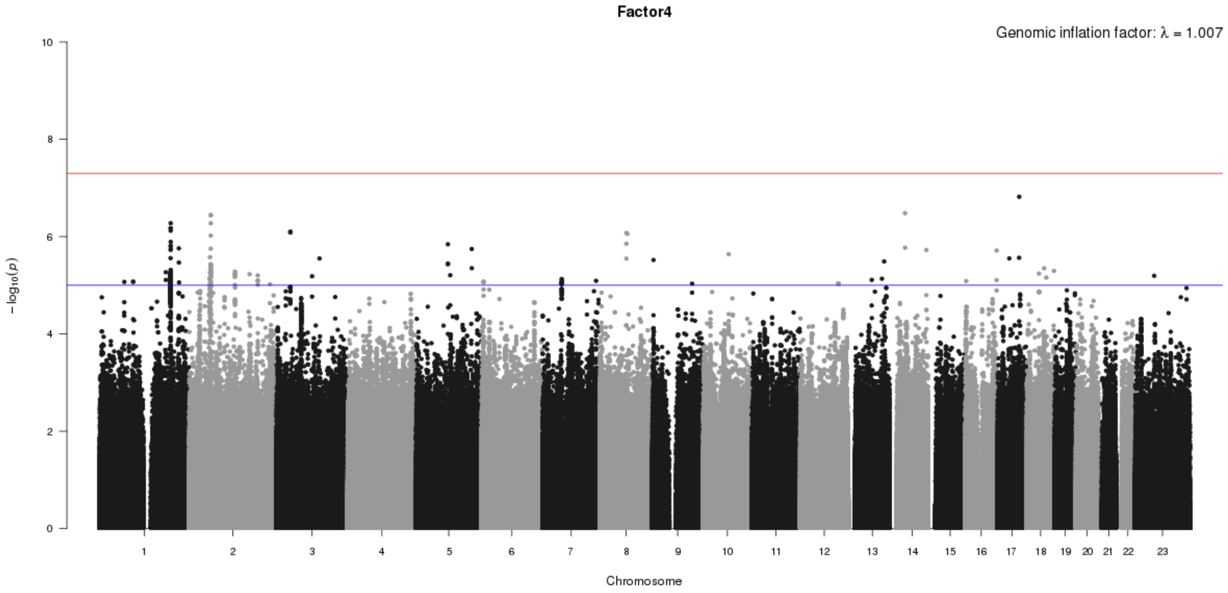
Factor4
Genomic inflation factor: λ = 1.007
−log10(p)
Chromosome
1 2 3 4 5 6 7 8 9 10 11 12 13 14 15 16 17 18 19 20 21 22 23

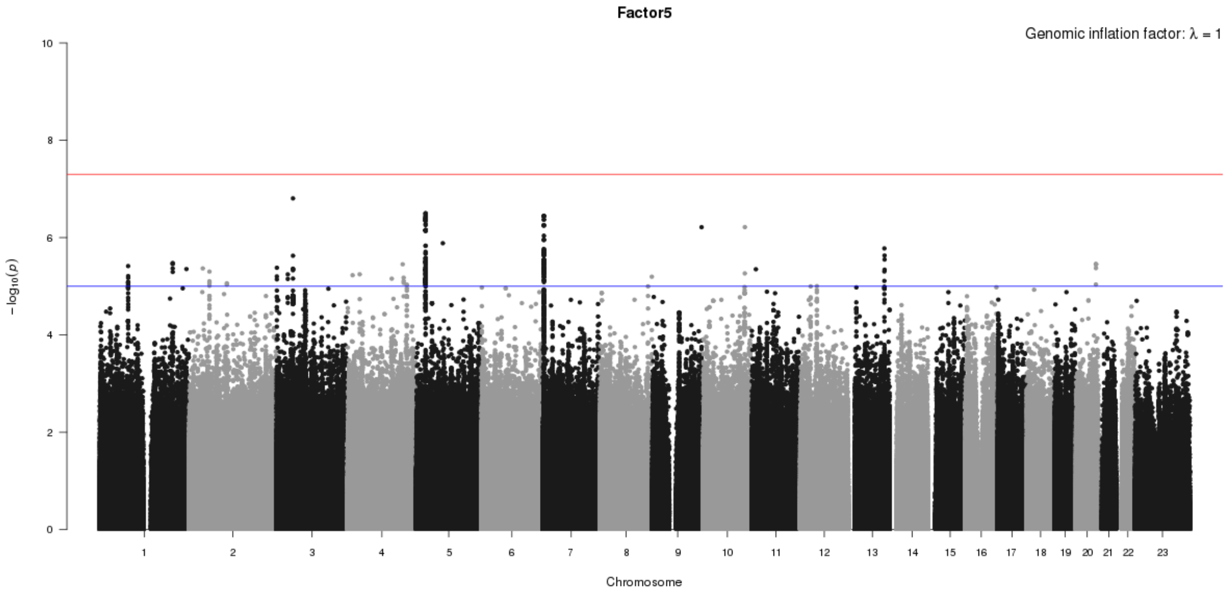

Factor5
Genomic inflation factor: λ = 1
−log10(p)
Chromosome

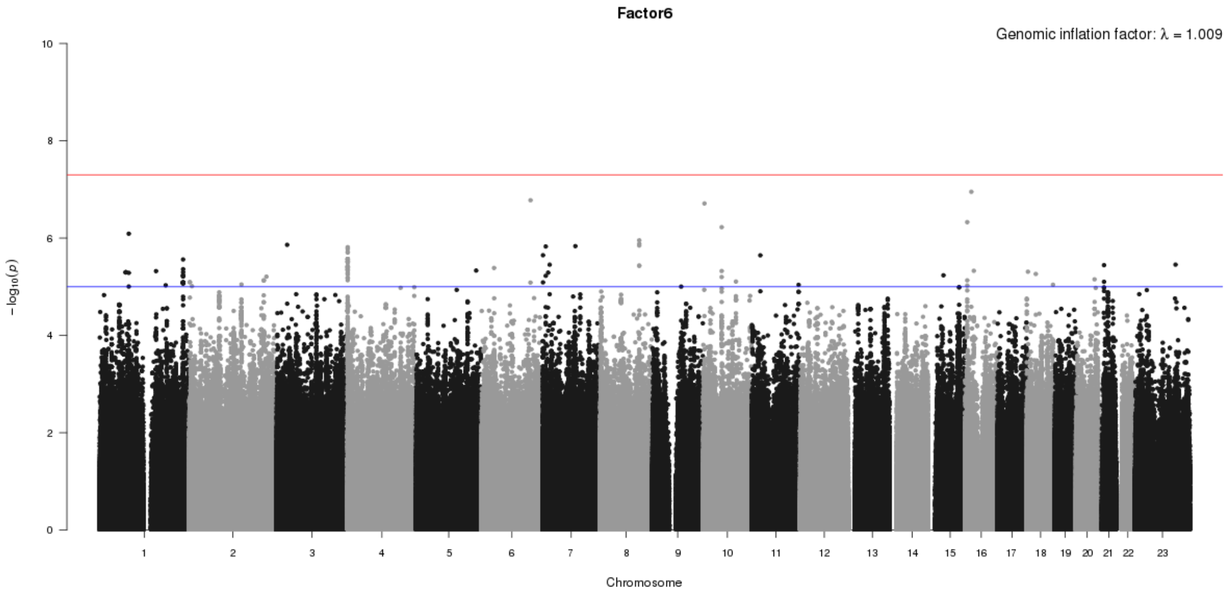

Factor6
Genomic inflation factor: λ = 1.009
−log10(p)
Chromosome

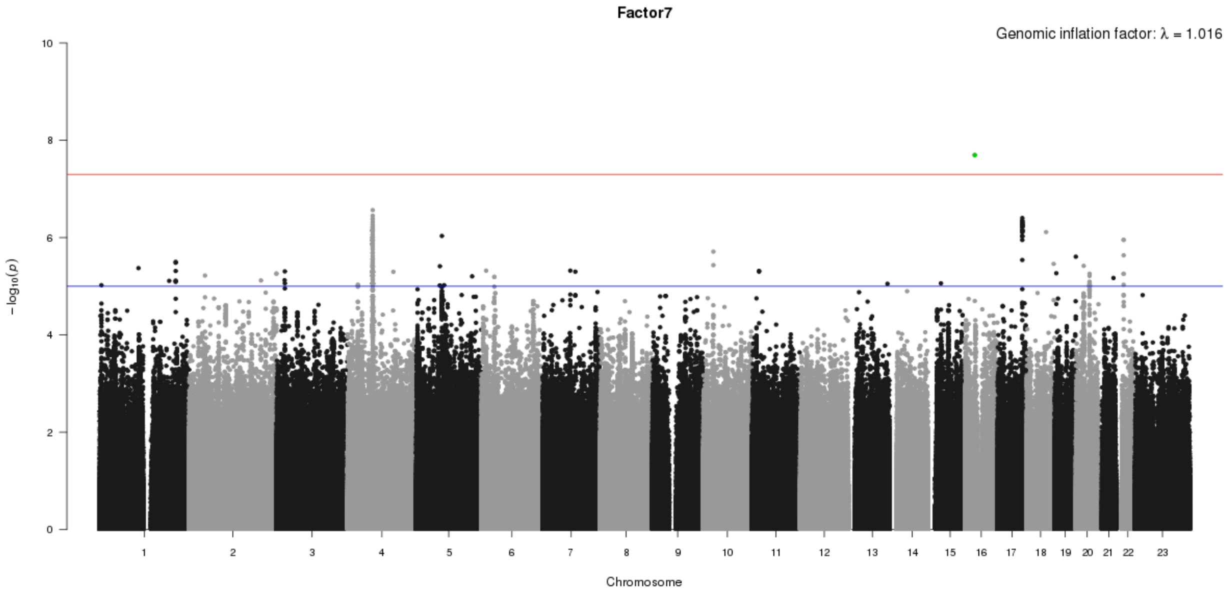
Factor7
Genomic inflation factor: λ = 1.016
10
8
6
4
2
0
−log10(p)
1
2
3
4
5
6
7
8
9
10
11
12
13
14
15
16
17
18
19
20
21
22
23
Chromosome

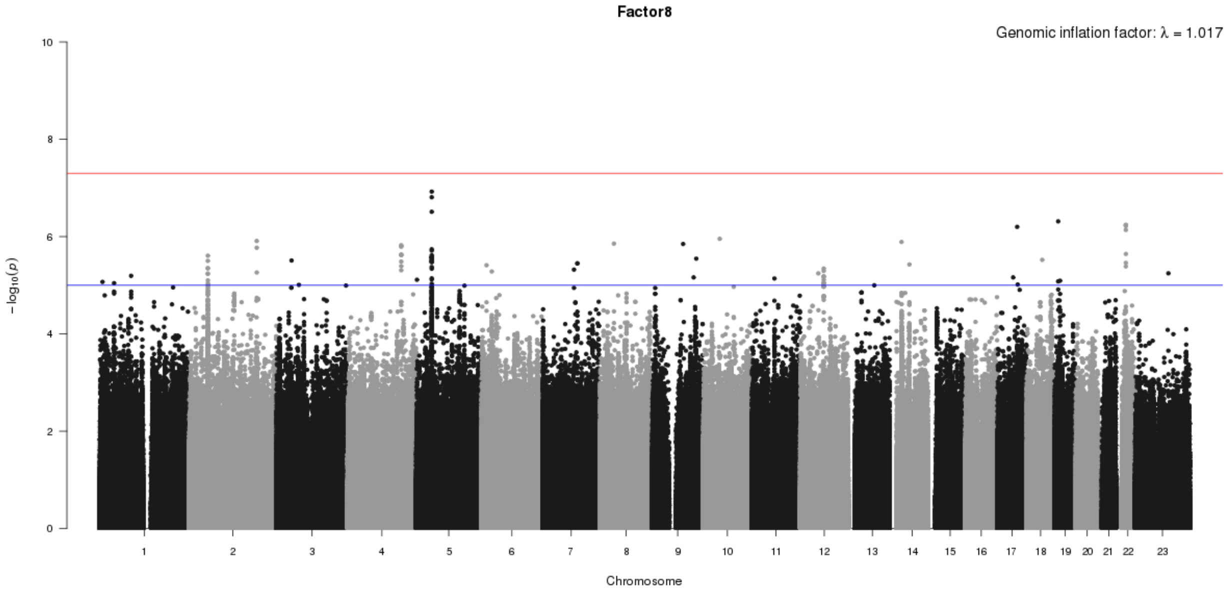
Factor8
Genomic inflation factor: λ = 1.017
10
8
6
4
2
0
−log10(p)
1
2
3
4
5
6
7
8
9
10
11
12
13
14
15
16
17
18
19
20
21
22
23
Chromosome

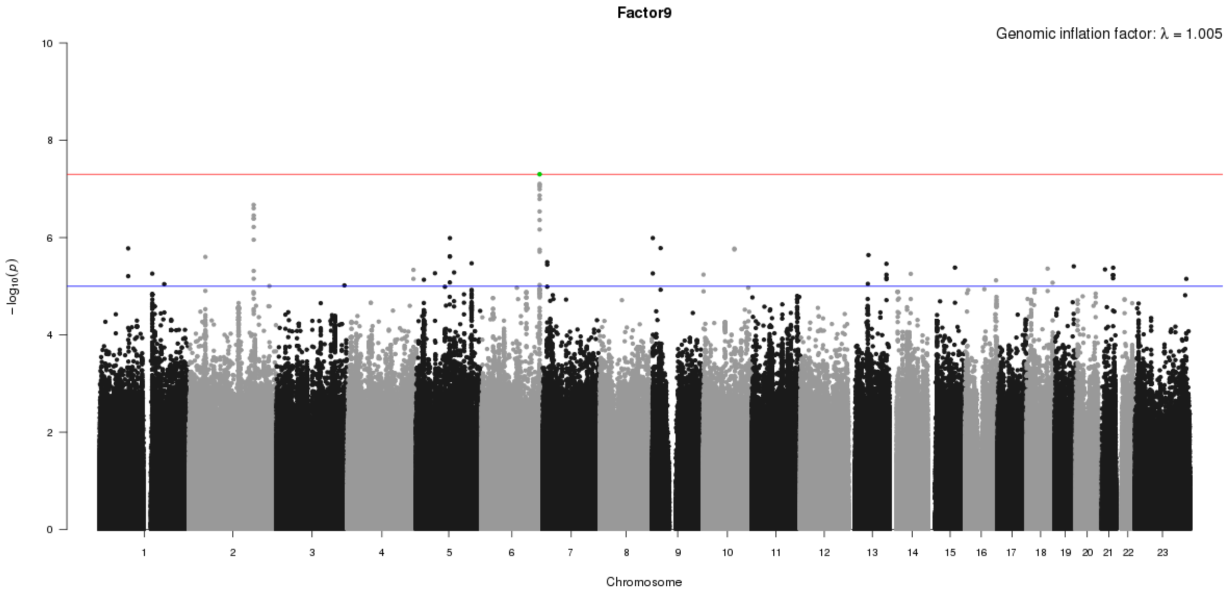
Factor9
Genomic inflation factor: λ = 1.005
$-\log_{10}(p)$
0
2
4
6
8
10
1
2
3
4
5
6
7
8
9
10
11
12
13
14
15
16
17
18
19
20
21
22
23
Chromosome

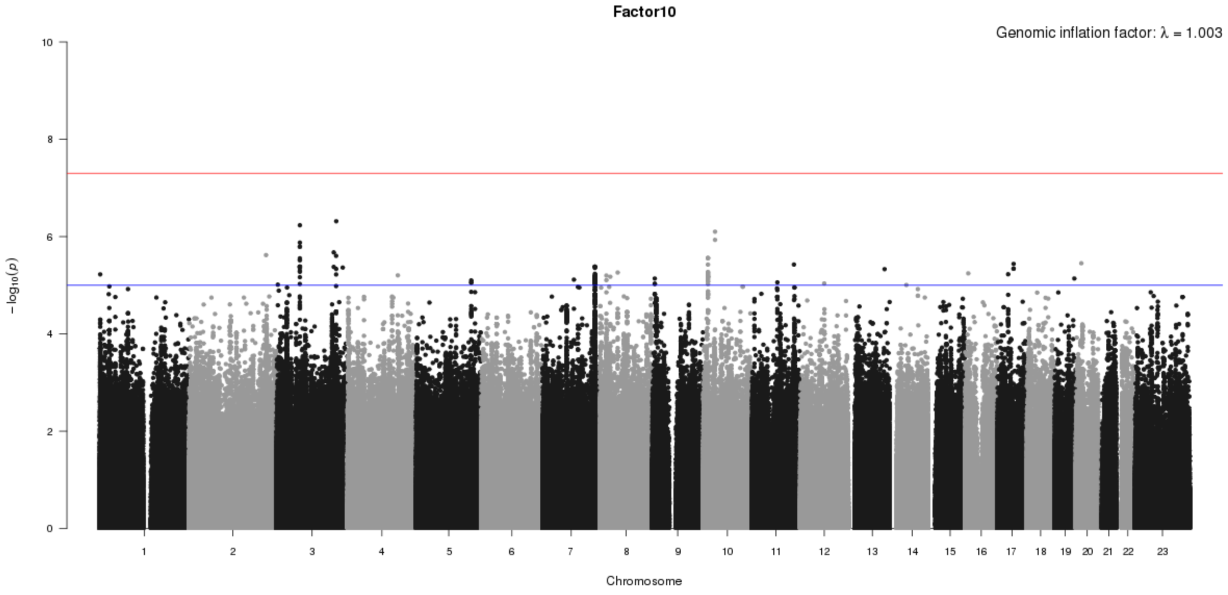
Factor10
Genomic inflation factor: λ = 1.003
$-\log_{10}(p)$
0
2
4
6
8
10
1
2
3
4
5
6
7
8
9
10
11
12
13
14
15
16
17
18
19
20
21
22
23
Chromosome

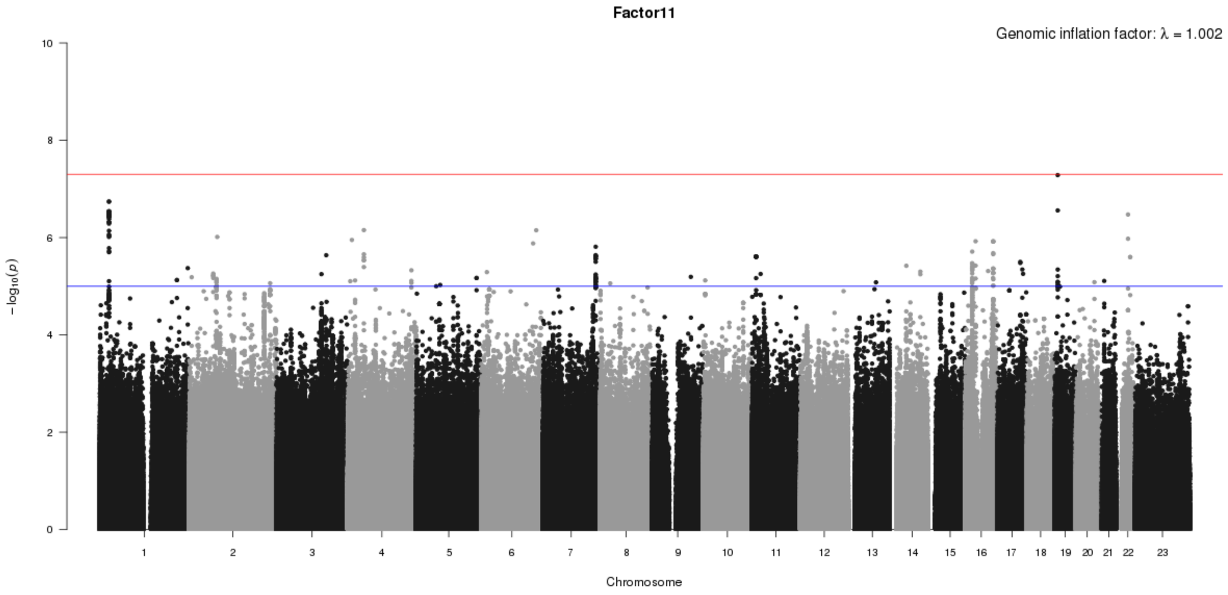
Factor11
Genomic inflation factor: λ = 1.002
−log10(p)
10
8
6
4
2
0
1 2 3 4 5 6 7 8 9 10 11 12 13 14 15 16 17 18 19 20 21 22 23
Chromosome

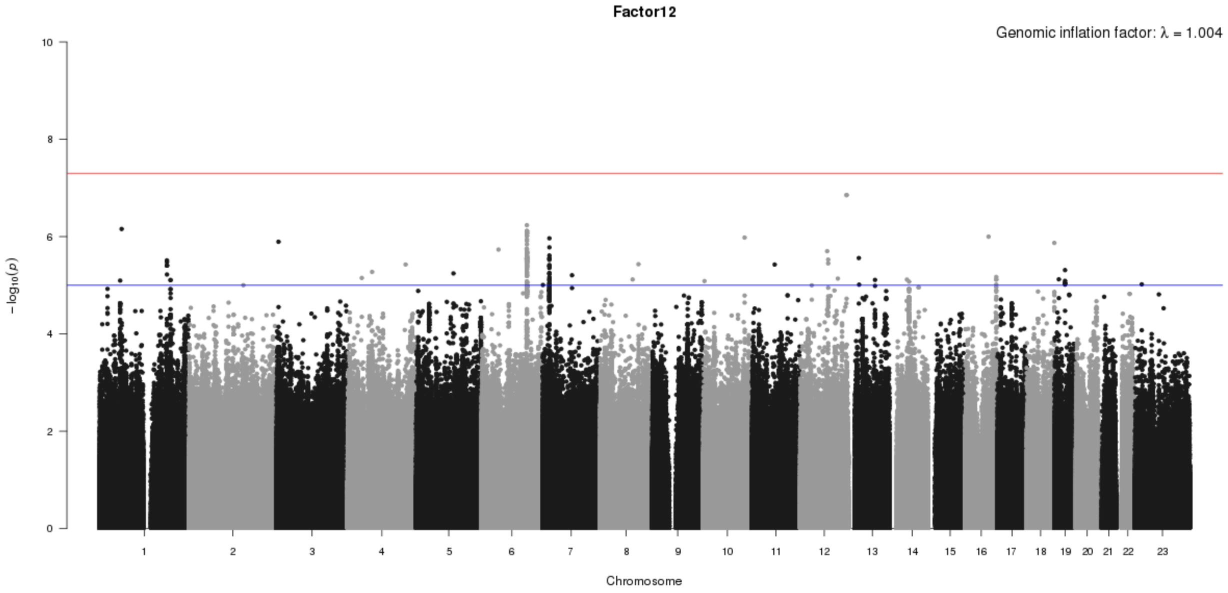
Factor12
Genomic inflation factor: λ = 1.004
−log10(p)
10
8
6
4
2
0
1 2 3 4 5 6 7 8 9 10 11 12 13 14 15 16 17 18 19 20 21 22 23
Chromosome

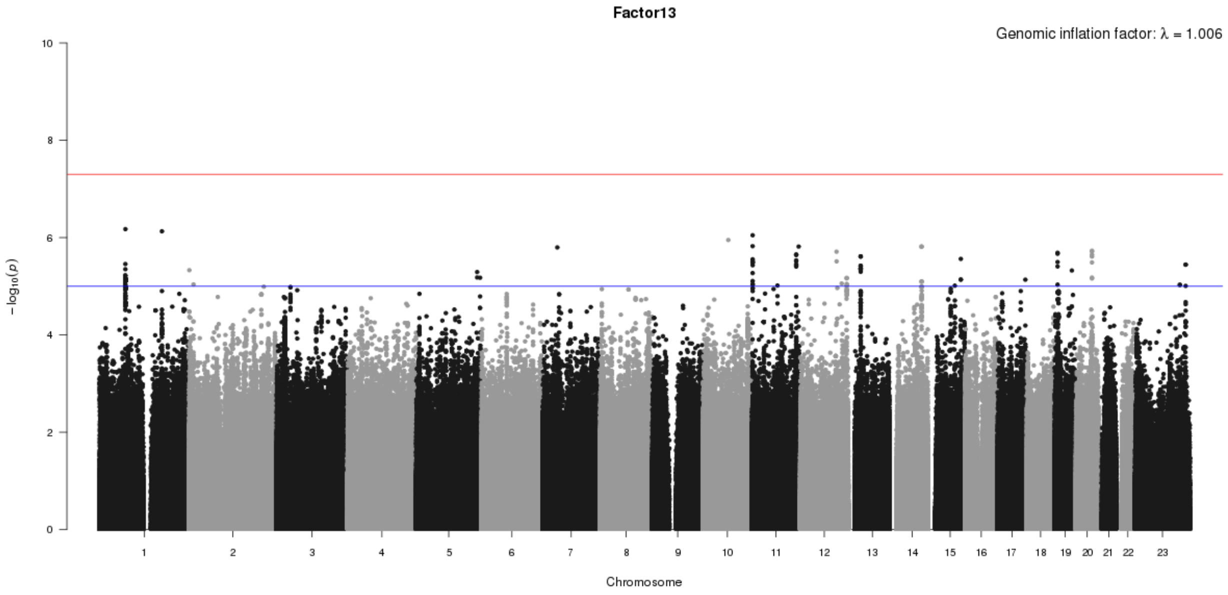
Factor13
Genomic inflation factor: λ = 1.006
−log10(p)
Chromosome
1
2
3
4
5
6
7
8
9
10
11
12
13
14
15
16
17
18
19
20
21
22
23

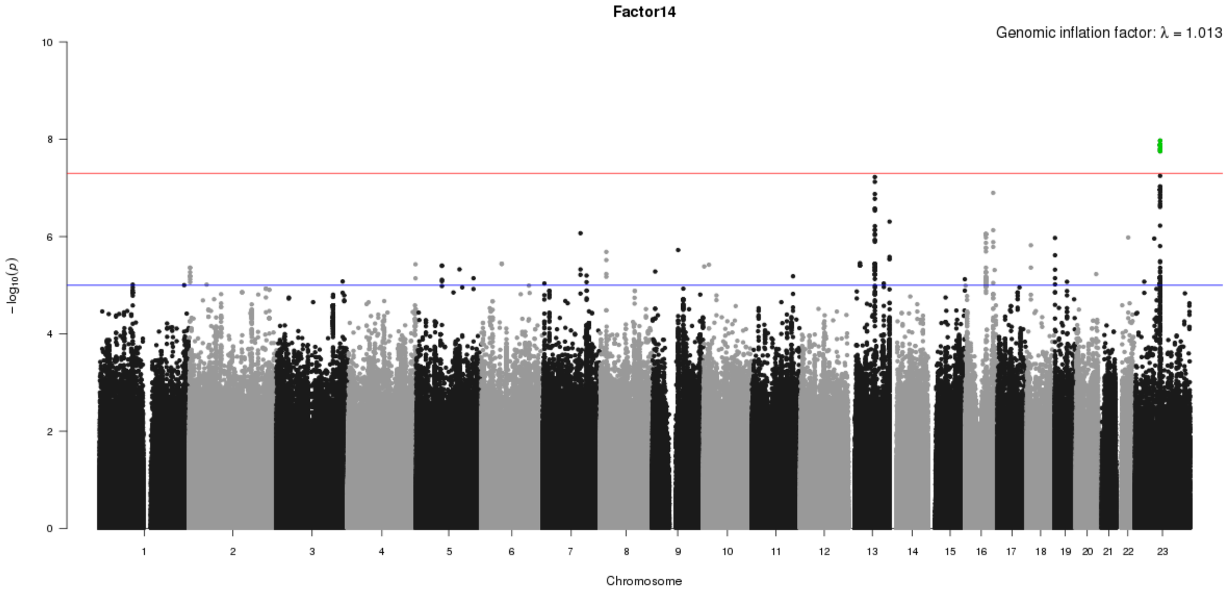
Factor14
Genomic inflation factor: λ = 1.013
−log10(p)
Chromosome
1
2
3
4
5
6
7
8
9
10
11
12
13
14
15
16
17
18
19
20
21
22
23

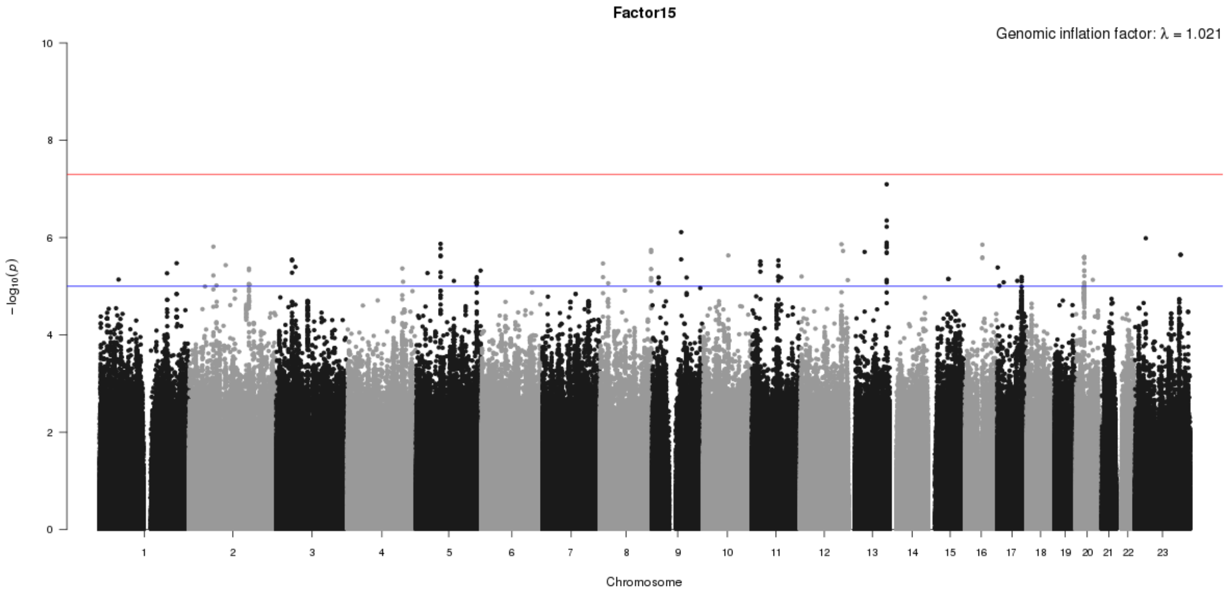

Factor15
Genomic inflation factor: λ = 1.021
−log10(p)
0
2
4
6
8
10
1
2
3
4
5
6
7
8
9
10
11
12
13
14
15
16
17
18
19
20
21
22
23
Chromosome

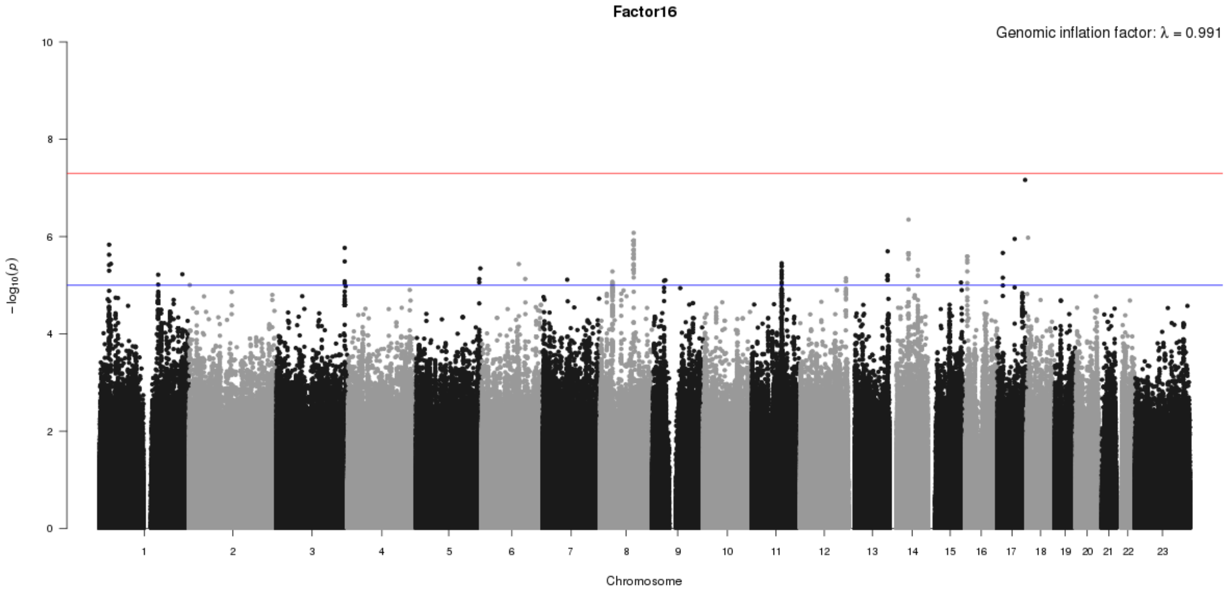

Factor16
Genomic inflation factor: λ = 0.991
−log10(p)
0
2
4
6
8
10
1
2
3
4
5
6
7
8
9
10
11
12
13
14
15
16
17
18
19
20
21
22
23
Chromosome

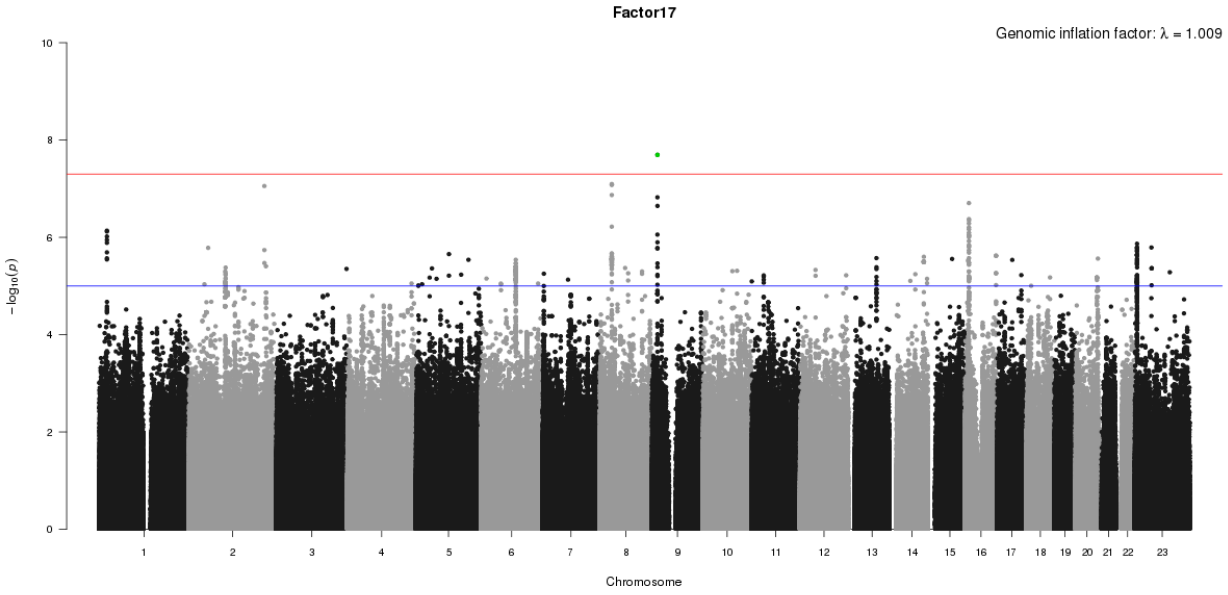

Factor17
Genomic inflation factor: λ = 1.009
$-\log_{10}(p)$
Chromosome

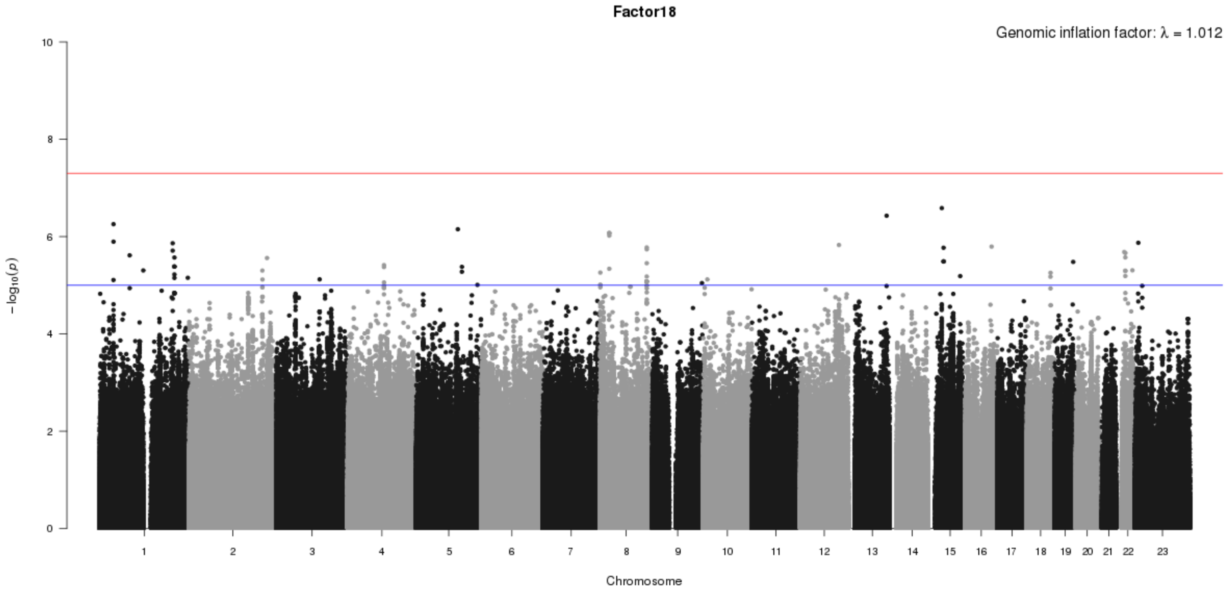

Factor18
Genomic inflation factor: λ = 1.012
$-\log_{10}(p)$
Chromosome

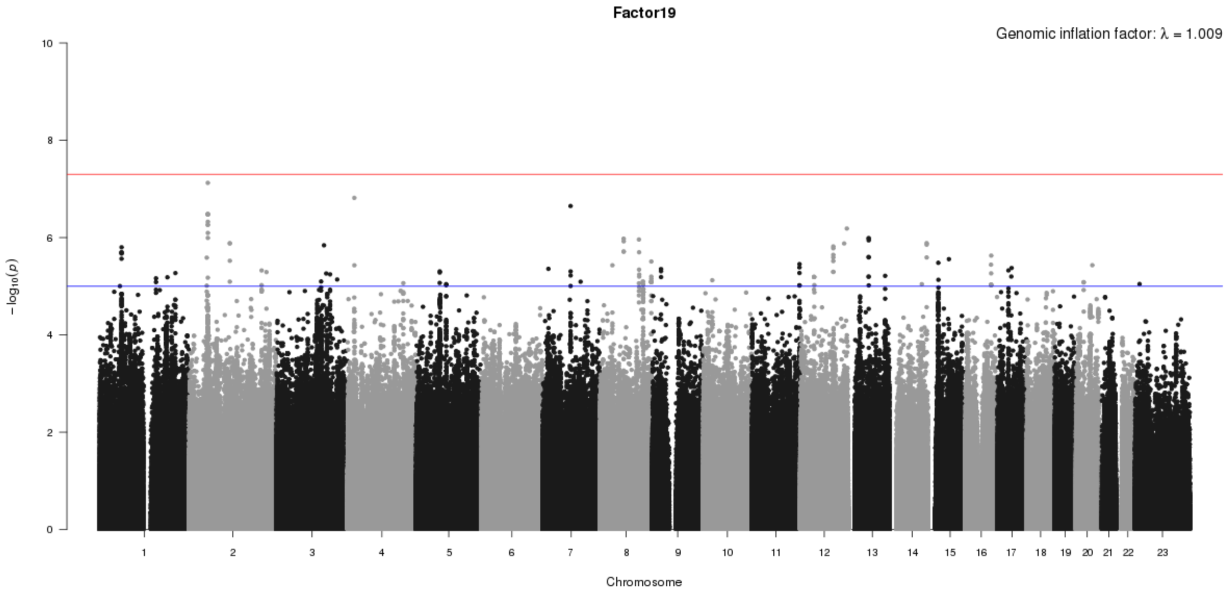
Factor19
Genomic inflation factor: λ = 1.009
10
8
6
4
2
0
$-\log_{10}(p)$
1
2
3
4
5
6
7
8
9
10
11
12
13
14
15
16
17
18
19
20
21
22
23
Chromosome

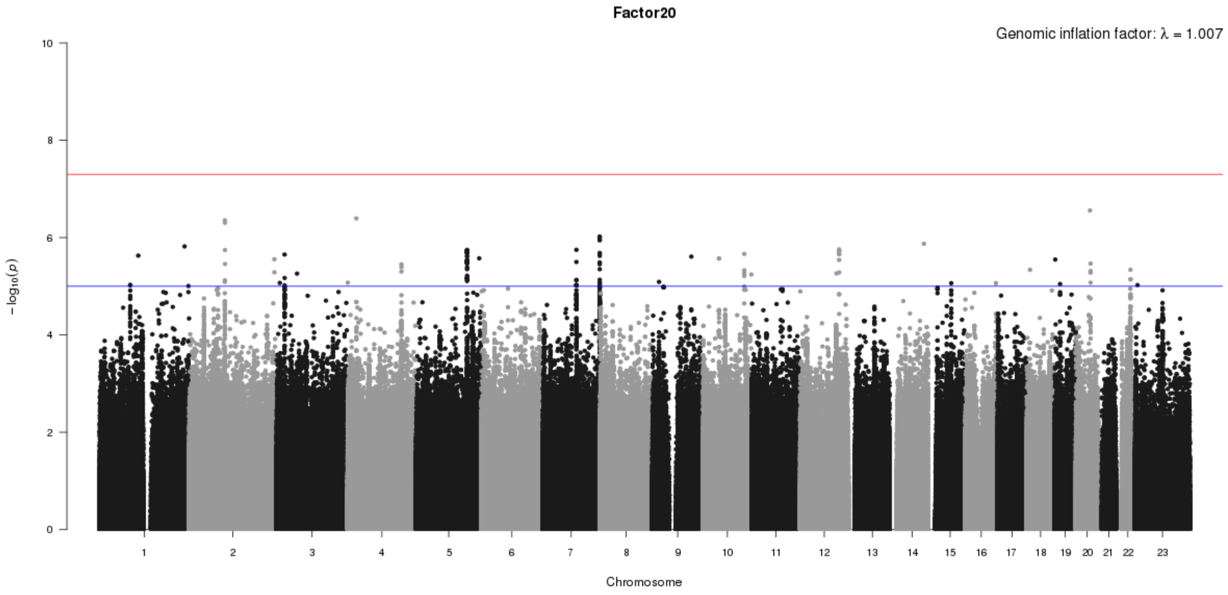
Factor20
Genomic inflation factor: λ = 1.007
10
8
6
4
2
0
$-\log_{10}(p)$
1
2
3
4
5
6
7
8
9
10
11
12
13
14
15
16
17
18
19
20
21
22
23
Chromosome

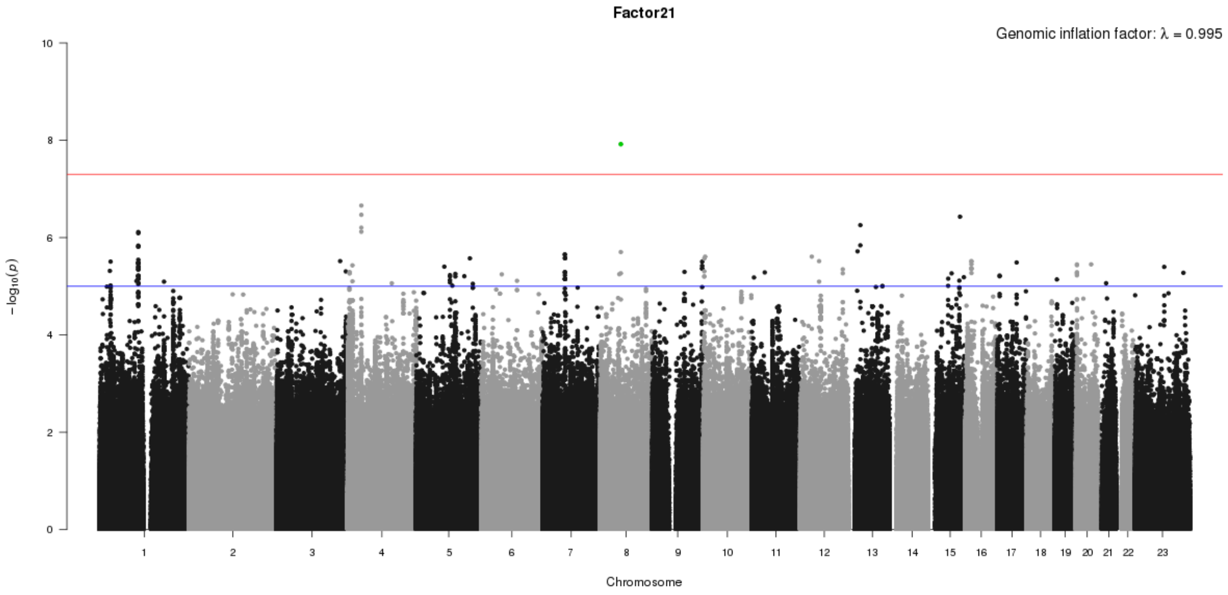
Factor21
Genomic inflation factor: λ = 0.995
−log10(p)
Chromosome

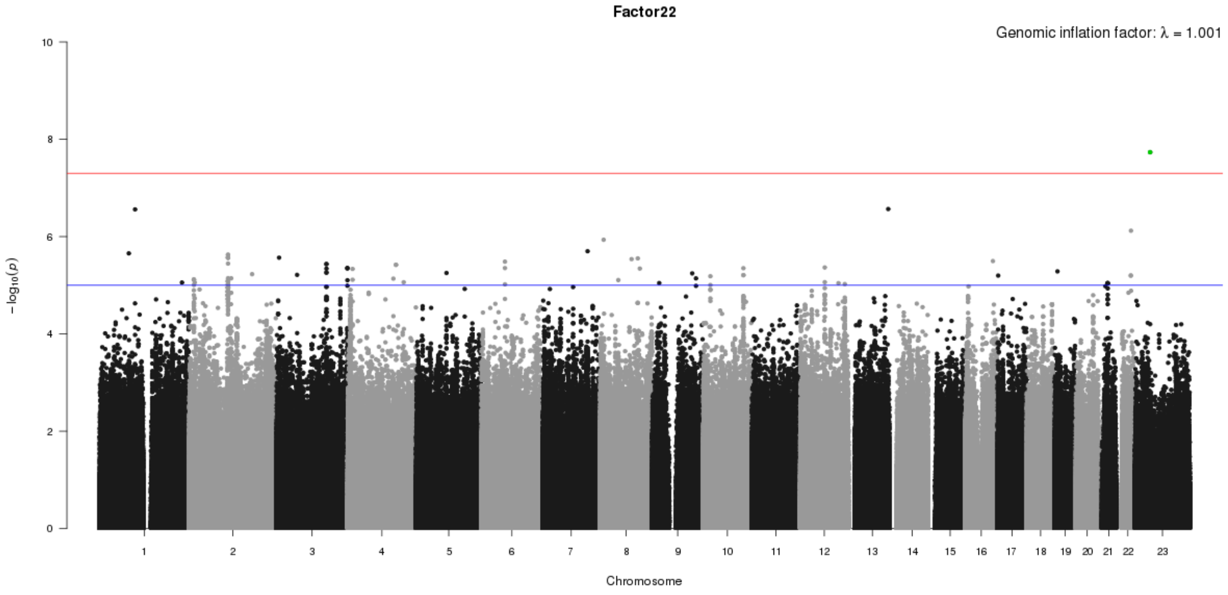
Factor22
Genomic inflation factor: λ = 1.001
−log10(p)
Chromosome

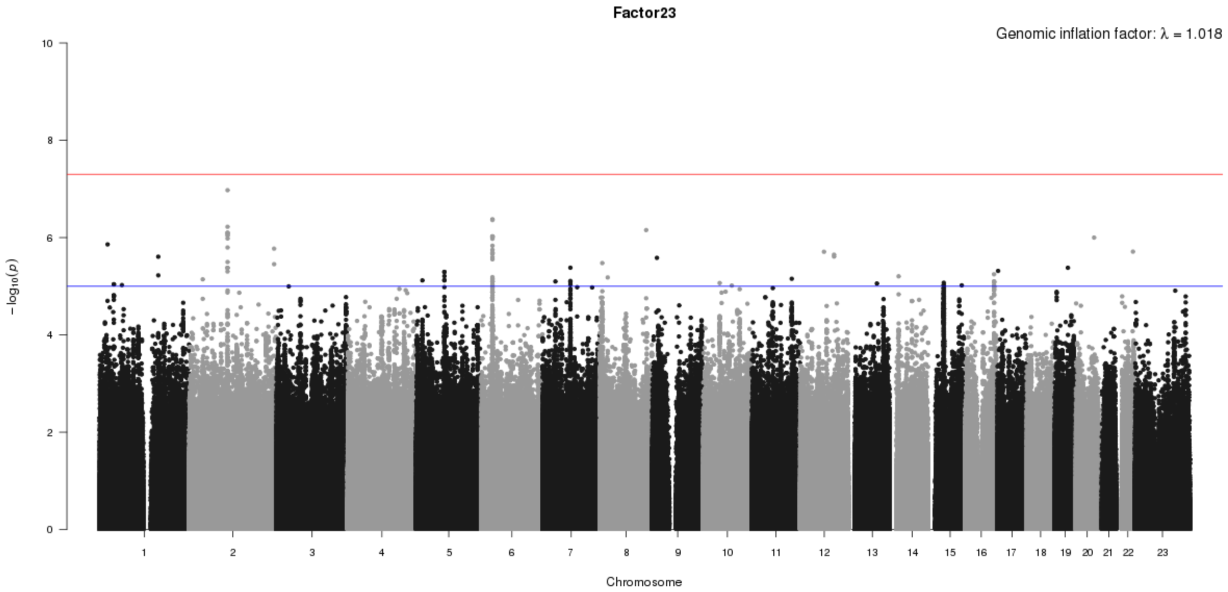

S3 Fig. Manhattan plots for the 23 factors showing all genotyped and imputed SNPs. Chromosomes are arranged in order along the x-axis. The y-axis shows the log base 10 p-value. Lines for p-value thresholds set at 5 x 10^{-8} for genome-wide significance and 5 x 10^{-6} for suggestive significance.
